# DNA barcoding a taxonomically complex hemiparasitic genus reveals deep divergence between ploidy levels but lack of species-level resolution

**DOI:** 10.1093/aobpla/ply026

**Published:** 2018-04-24

**Authors:** Xumei Wang, Galina Gussarova, Markus Ruhsam, Natasha de Vere, Chris Metherell, Peter M Hollingsworth, Alex D Twyford

**Affiliations:** 1 School of Pharmacy, Xi’an Jiaotong University, Xi’an, P.R. China; 2 Tromsø University Museum, UiT The Arctic University of Norway, Langnes, Tromsø, Norway; 3 CEES-Centre for Ecological and Evolutionary Synthesis, Department of Biosciences, University of Oslo, Blindern, Oslo, Norway; 4 Department of Botany, Faculty of Biology, St Petersburg State University, Universitetskaya nab., Russia; 5 Royal Botanic Garden Edinburgh, Edinburgh, UK; 6 National Botanic Garden of Wales, Llanarthne, Carmarthenshire, UK; 7 Institute of Biological, Environmental & Rural Sciences (IBERS), Aberystwyth University, Penglais, Aberystwyth, Ceredigion, UK; 8 57 Walton Road, Bristol, UK; 9 University of Edinburgh, Institute of Evolutionary Biology, Edinburgh, UK

**Keywords:** British flora, DNA barcoding, *Euphrasia*, Orobanchaceae, phylogeny, polyploidy, taxonomic complexity

## Abstract

DNA barcoding is emerging as a useful tool not only for species identification but also for studying evolutionary and ecological processes. Although plant DNA barcodes do not always provide species-level resolution, the generation of large DNA barcode data sets can provide insights into the mechanisms underlying the generation of species diversity. Here, we study evolutionary processes in taxonomically complex British *Euphrasia* (Orobanchaceae), a group with multiple ploidy levels, frequent self-fertilization, young species divergence and widespread hybridization. We use a phylogenetic approach to investigate the colonization history of British *Euphrasia*, followed by a DNA barcoding survey and population genetic analyses to reveal the causes of shared sequence variation. Phylogenetic analysis shows *Euphrasia* have colonized Britain from mainland Europe on multiple occasions. DNA barcoding reveals that no British *Euphrasia* species has a consistent diagnostic sequence profile, and instead, plastid haplotypes are either widespread across species, or are population specific. The partitioning of nuclear genetic variation suggests differences in ploidy act as a barrier to gene exchange, while the divergence between diploid and tetraploid ITS sequences supports the polyploids being allotetraploid in origin. Overall, these results show that even when lacking species-level resolution, analyses of DNA barcoding data can reveal evolutionary patterns in taxonomically complex genera.

## Teaser

The sequencing of short standardized pieces of DNA (DNA barcodes) is commonly used to tell species apart. This approach is thought to be of limited value in complex plant groups where species have evolved recently. We use DNA barcoding in taxonomically complex British *Euphrasia*, and while we find no species with a diagnostic sequence profile, we do see evolutionary patterns, such as divergence between two different species groups with different chromosome numbers. This shows DNA barcoding can be useful for studying evolutionary patterns in complex plant groups.

## Introduction

DNA barcoding is a valuable tool for discriminating among species, and these data often give insights into identity that are overlooked based on morphology alone (Hebert and Gregory 2005). DNA barcoding relies on sequencing a small set of gene regions (such as the core plant DNA barcode *rbcL* + *matK*, often supplemented with *ITS* and other regions), and using these data for species identification (CBOL Plant Working Group 2009). Successful applications of DNA barcoding include species discovery, reconstructing historical vegetation types from frozen sediments, surveying environmental variation and many other research topics (reviewed in Hollingsworth *et al.* 2016). However, there are numerous reports of taxon groups where the standard DNA barcode sequences do not provide exact plant species identification, and where DNA barcode sequences are shared among related species (Spooner 2009; Percy *et al.* 2014; Zarrei *et al.* 2015; Yan *et al.* 2015a, b). Even in these cases, however, the generation of large data sets of DNA sequences from multiple individuals of multiple species can shed light onto evolutionary relationships and patterns of divergence, without a need for the barcode markers to track species boundaries.

Postglacial species radiations of taxonomically complex groups in Northern Europe are a case where we may not expect a clear cut-off between intraspecific variation and interspecific divergence and thus DNA barcoding may provide limited discriminatory power. Such postglacial groups include the *Arabidopsis arenosa* complex (Schmickl *et al.* 2012), *Cerastium* (Brysting *et al.* 2007), *Epipactis* (Squirrell *et al.* 2002) and *Galium* (Kolář *et al.* 2015). Despite this complexity, DNA barcoding may still be valuable if used to identify evolutionary and ecological processes that result in shared sequence variation. For example, many postglacial taxa are characterized by a combination of: (i) recent postglacial speciation, (ii) extensive hybridization, (iii) frequent self-fertilization, (iv) divergence involving polyploidy. Our expectation is that factors (i) + (ii) will cause DNA barcode sequences to be shared among geographically proximate taxa, while (iii) will cause barcodes to be population rather than species specific (Hollingsworth *et al.* 2011; Naciri *et al.* 2012). Factor (iv), polyploidy, will manifest as shared variation between recent polyploids and their parental progenitors, or deep allelic divergence in older polyploid groups, where ploidy acts as a reproductive isolating barrier and allows congeneric taxa to accumulate genetic differences.

One example of a taxonomically challenging group showing postglacial divergence is British *Euphrasia* species (Ennos *et al.* 2005). This group of 19 taxa is renowned for their difficult species identification, and at present only a handful of experts can identify these species in the field. Morphological species identification is difficult due to their small stature, combined with species being defined by a complex suite of overlapping characters (Yeo 1978). They are also generalist hemiparasites and thus phenotypes are plastic and may depend upon host quality (Svensson and Carlsson 2004). DNA barcode-based identification could partly resolve these identification issues, and lead to a greater understanding of species diversity and distributions in this under-recorded group. This is particularly important as a number of *Euphrasia* species are critically rare and of conservation concern, while others are ecological specialists that are useful indicators of habitat type (French *et al.* 2008). More generally, DNA barcoding data could reveal the processes structuring genetic diversity and those that are responsible for recent speciation.

Previous broad-scale surveys of *Euphrasia* using amplified fragment length polymorphisms (AFLPs) and microsatellites have shown a significant proportion of genetic variation is partitioned between two ploidy groups (diploids and tetraploids), and by species, despite extensive hybridization (French *et al.* 2008). Here, we follow-on from this population genetic study by using DNA sequence data to investigate the processes underlying the regional assembly of British *Euphrasia* diversity. Our first aim is to understand whether British endemic *Euphrasia* are a product of speciation within a single clade, or if speciation has occurred within multiple groups of genetically diverse European relatives. We address this question of regional assembly by placing British *Euphrasia* species in the context of a global *Euphrasia* phylogeny. In the light of our phylogenetic analysis, our second aim is to deploy DNA barcoding across a large British sample set to characterize patterns of genetic diversity and the potential factors underlying shared sequence variation. The combination of our two data sets also sheds light on the role that polyploidy plays in shaping genetic diversity in *Euphrasia*, with our phylogenetic analysis revealing whether polyploidization has occurred recently in British taxa (or occurred before colonizing the UK), while our DNA barcoding shows whether ploidy differences are a barrier to gene exchange. Overall, these results are used to improve our understanding of the evolution of a complex regional plant assembly, and to test the efficacy of DNA barcoding for studying species-level variation in a taxonomically complex group.

## Methods

### Specimen sampling

**Figure 1.**
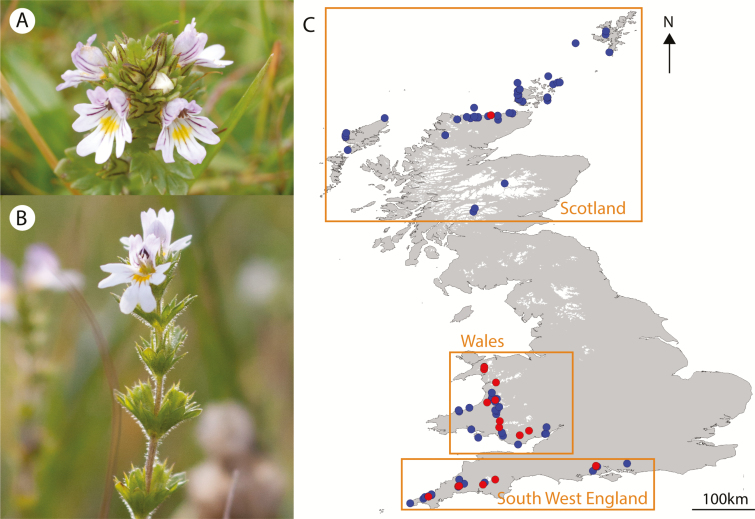
*Euphrasia* samples used in this study. (A) Tetraploid British *Euphrasia* (here *E. arctica*) have glabrous leaves sometimes with sparse short eglandular hairs or bristles. (B) Diploid British *Euphrasia* have long glandular hairs. (C) Collection sites of *Euphrasia* DNA samples. Diploids are shown in red, tetraploids in blue. Orange boxes correspond to the three broad sampling areas. Photo credits: Alex Twyford, Max Brown.

The 19 currently recognized British *Euphrasia* species are all annual, selfing or mixed-mating small herbaceous plants, which occur in a range of habitats including coastal turf, chalk downland, mountain ridges and heather moorland (French *et al.* 2004). The species can be divided into two groups, glabrous or short eglandular hairy tetraploids (15 species, Fig. 1A), or long glandular hairy diploids (4 species, Fig. 1B). Our sampling includes representatives of all British species (Fig. 1C; Supporting Information—Table S1). Samples were collected in South West England and Wales to allow us to include mixed populations of diploids and tetraploids, early generation diploid × tetraploid hybrids, and two diploid hybrid species hypothesized to be derived from diploid × tetraploid crosses (*E. vigursii*, parentage: *E. rostkoviana* × *E. micrantha*; *E. rivularis*, parentage: *E. anglica* × *E. micrantha*; Yeo 1956). Samples from Scotland allows us to sample complex tetraploid taxa and tetraploid hybrids, plus scarcer Scottish diploids. Our sampling scheme investigated range-wide variation by targeting many taxa and populations, with a focus on collecting multiple species in areas of sympatry. We chose not to include detailed intrapopulation sampling because prior work has shown low intrapopulation diversity, with populations frequently fixed for a given allele (French *et al.* 2008). All samples collected prior to 2012 were identified by former *Euphrasia* referee Alan Silverside, while recent samples were identified by current referee Chris Metherell.

For our molecular phylogenetic analysis aimed at understanding the colonization history of British *Euphrasia*, we expanded the sampling in the phylogeny of the genus by Gussarova *et al.* (2008) to include a detailed sample of British taxa. The previous analysis included 41 taxa for the nuclear ribosomal internal transcribed spacer (ITS), and 50 taxa for plastid DNA (Gussarova *et al.* 2008). We sequenced samples to match the previous data matrix, which included: the *trnL* intron (Taberlet *et al.* 1991), intergenic spacers *atpB-rbcL* (Hodges and Arnold 1994) and *trnL-trnF* (Taberlet *et al.* 1991), and *ITS* (White *et al.* 1990).

For our population-level DNA barcoding study, we analysed a total of 133 individuals, with 106 samples representing 19 species, as well as 27 samples from 14 putative hybrid taxa. We sequenced samples for the core plant DNA barcoding loci, *mat*K and *rbc*L (CBOL Plant Working Group 2009), as well as partial sequences of *ITS* (*ITS2*), which has been suggested to be incorporated into the core DNA barcode (China Plant BOL Group 2011). We also followed the recommendation of Hollingsworth *et al.* (2011), to add a non-coding gene to our set of plastid loci to help resolve recent haplotype divergence. We used *rpl32*-*trnL*^*UAG*^, which has been informative in prior population studies of *Euphrasia* (Stone 2013).

### DNA extraction, PCR amplification and sequencing

DNA was extracted from silica-dried tissue using the DNeasy Plant Mini Kit (Qiagen, Hilden, Germany) following the manufacturer’s protocol, but with an extended incubation of 1 h at 65 °C. These DNA samples were added to existing DNA extractions of 68 individuals from French *et al.* (2008).

We performed PCRs in 10 μL reactions, with DNA amplification and PCR conditions for each primer given in Supporting Information—Table S2. We visualized PCR products on a 1 % agarose gel, with 5 μL of PCR product purified for sequencing with ExoSAP-IT (USB Corporation, Cleveland, OH, USA) using standard protocols. Sequencing was performed in 10 μL reactions containing 1.5 μL 5× BigDye buffer (Life Technologies, Carlsbad, CA, USA), 0.88 μL BigDye enhancing buffer BD × 64 (MCLAB, San Francisco, CA, USA), 0.125 μL BigDye v3.1 (Life Technologies), 0.32 μM primer and 1 μM of purified PCR product. We sequenced PCR products on the ABI 3730 DNA Analyser (Applied Biosystems, Foster City, CA, USA) at Edinburgh Genomics. In addition to these newly generated sequences, a subset of sequences were generated as part of the effort to DNA barcode the UK Flora, and followed a different set of protocols, detailed in de Vere *et al.* (2012).

We assembled, manually edited and aligned sequences using Geneious v. 8 (Biomatters, Auckland, New Zealand). We scored indels as unordered binary characters and appended them to the matrices. We used gap coding as implemented in Gapcoder (Young and Healy 2003), with indels treated as point mutations and equally weighted with other mutations.

### Phylogenetic analysis of global *Euphrasia*

We used Bayesian phylogenetic analyses in MrBayes v. 3.1.2 (Huelsenbeck and Ronquist 2001) to infer species relationships and broad-scale patterns of colonization. Our analyses used a sequence matrix that included our newly sampled British taxa in addition to previous global *Euphrasia* samples from Gussarova *et al.* (2008). We selected the best fitting model of nucleotide substitution using the Akaike Information Criterion (AIC) with an empirical correction for small sample sizes implemented in MrAIC (Nylander 2004). Using GTR + G as the best model for the plastid data set and SYM + G for the ITS data set we ran two sets of four Markov Chain Monte Carlo (MCMC) runs for 5000000 generations. Indels were included as a separate partition with a restriction site (binary) model. We sampled every 1000th generation and discarded the first 25 % as burn-in. We confirmed chain convergence by observing the average standard deviation of split frequencies and by plotting parameter values in Tracer v. 1.6 (Rambaut and Drummond 2013). The alignment and trees are deposited in TreeBase under accession number 22492 (https://treebase.org).

### DNA barcoding survey of British taxa

We examined patterns of sequence variation using a range of population genetic methods. We investigated the amount of sequence diversity across species using descriptive statistics, and then tested the cohesiveness of taxa using analysis of molecular variance (AMOVA) and related methods. Analyses were performed separately on ITS2 and a concatenated matrix of either all sampled plastid loci, or just the core DNA barcode loci. For plastid data, haplotypes were determined from nucleotide substitutions and indels of the aligned sequences. Basic population genetic statistics were performed in Arlequin Version 3.0 (Excoffier and Lischer 2010), and this included the number of haplotypes, as well as hierarchical AMOVA in groups according to: (i) ploidy level (diploid vs. tetraploid); (ii) geographic regions (Wales, England, Scotland); (iii) species. Analysis of molecular variance was performed on all taxa, and repeated for ploidy levels and geographic regions on a data set only including confirmed species (i.e. excluding hybrids). Sequence diversity and divergence statistics were estimated with DnaSP (Librado and Rozas 2009), which included: average nucleotide diversity across taxa (π), Watterson’s *θ* (per site), Tajima’s *D* and divergence between ploidy levels (*D*_XY_).

Genetic divergence among sampling localities was explored with spatial analysis of molecular variance (SAMOVA; Dupanloup *et al.* 2002), implemented in SPADS v.1.0 (Dellicour and Mardulyn 2014). Spatial analysis of molecular variance maximizes the proportion of genetic variance due to differences among populations (*F*_CT_) for a given number of genetic clusters (*K*-value). We considered the best grouping to have the highest *F*_CT_ value after 100 repetitions. This analysis investigated interspecific differentiation, thus only used species samples, excluding hybrids.

The relationships between haplotypes were inferred by constructing median-joining networks (Bandelt *et al.* 1999) with the program NETWORK v.4.6.1.1 (available at http://www.fluxus-engineering.com/), treating gaps as single evolutionary events.

## Results

### Global phylogenetic analysis

**Figure 2.**
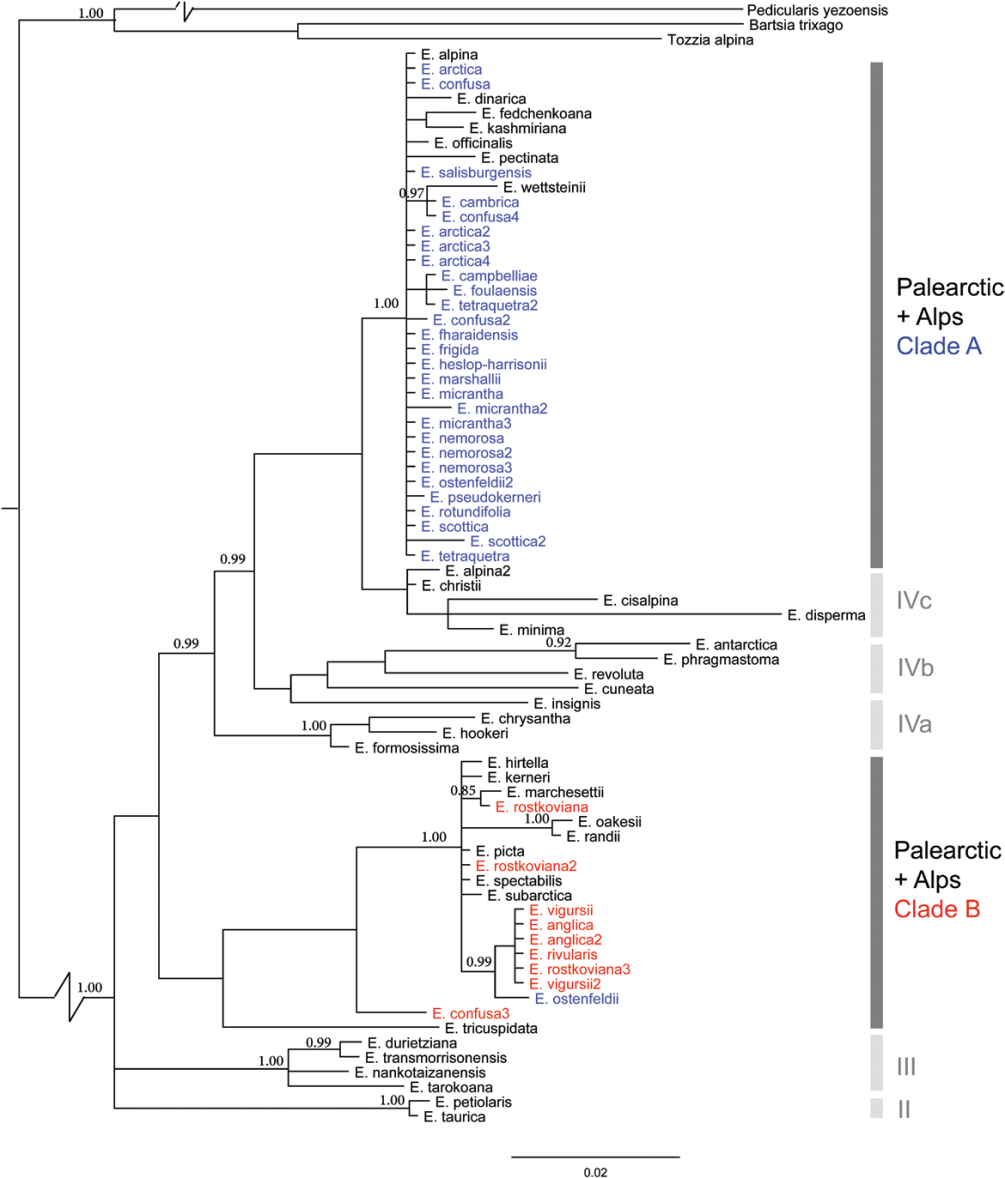
Majority rule consensus phylogeny of *Euphrasia* inferred from the ITS region using MrBayes. Posterior probabilities >0.85 are indicated. Individuals are coloured by ploidy and geography: British diploids (red), British tetraploids (blue), other geographic areas (black). Clade A and Clade B correspond to the main study groups, with additional clades corresponding to Gussarova *et al.* (2008) also marked: II northern tetraploids; III Taiwan; IVa South American/Tasmanian; IVb complex (South American, New Zealand, Japan); IVc Alpine European.

The final ITS alignment was 638 bp in length, for a total of 76 *Euphrasia* samples, including those from Gussarova *et al.* (2008). Our broad-scale global *Euphrasia* phylogenetic analysis performed using MrBayes gave meaningful clusters of species, though the tree topology was generally poorly supported with many polytomies (Fig. 2). British taxa predominantly belonged to two clusters: a tetraploid clade of Holarctic taxa from Sect. *Euphrasia* (posterior probability support, pp = 1.00, Clade A, Fig. 2), and a well-supported geographically restricted Palearctic diploid lineage (pp = 1.0, Clade B, Fig. 2). The tetraploid clade included a mix of British and European taxa, and is sister to a mixed clade of alpine diploid species and tetraploid *E. minima* (Clade IVc, Fig. 2). The diploid clade includes British diploids *E. anglica*, *E. rivularis*, *E. rostkoviana*, *E. vigursii* and European relatives (diploids or taxa without chromosome counts). The only non-diploid in the clade is one individual of tetraploid British *E. ostenfeldii*, which appears to be correctly identified and thus may have captured the diploid ITS variant through hybridization. Overall, terminal branches of the tree are short, indicative of limited divergence. The only exception was the long branch of *E. disperma* from New Zealand, a result seen in previous Bayesian analyses (cf. Gussarova *et al.* 2008, Fig. 2) but not in parsimony analyses, where it clusters together with the other southern hemisphere species on a shorter branch (Gussarova *et al.* 2008).

**Figure 3.**
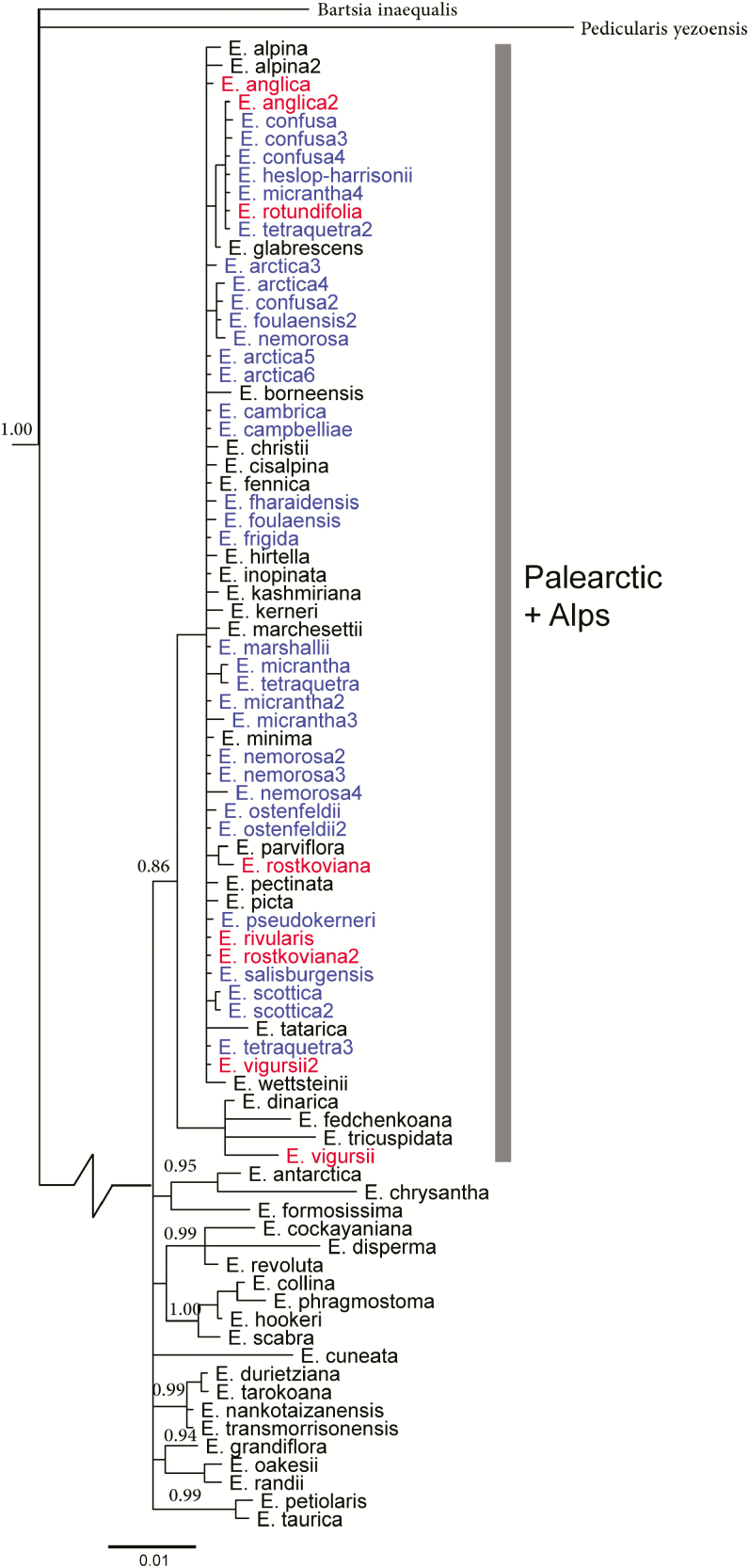
Majority rule consensus phylogeny of *Euphrasia* inferred from a concatenation of plastid *trn*L intron, *trn*L-*trn*F and *atp*B-*rbc*L using MrBayes. Posterior probabilities >0.85 are indicated, and British diploids (red) and British tetraploids (blue) are coloured.

The final concatenated plastid alignment was 1692 bp in length, for a total of 82 *Euphrasia* samples, including those from Gussarova *et al.* (2008). This alignment included the *trn*L intron (517 bp, 73 variable sites), *trn*L-*trn*F (420 bp, 85 variable sites) and *atp*B-*rbc*L (754 bp, 89 variable sites). The plastid tree (Fig. 3) recovered the geographic clades reported in Gussarova *et al.* (2008). All diploid and tetraploid British samples possessed plastid haplotypes from the broad Palearctic clade, which also includes *E. borneensis* (Borneo) and *E. fedtschenkoana* (Tian Shan). This clade received moderate support in our analysis (pp = 0.85). While partially informative of broad-scale relationships, most terminal branches were extremely short, and gave no information on interspecific relationships.

### UK DNA barcoding

#### ITS diversity.

The final ITS2 alignment contained 130 individuals representative of all 19 British taxa, and was 380 bp in length. Only two samples (of *E. scottica*) presented double peaks, and were excluded from analyses. Overall diversity across taxa was modest, with a nucleotide diversity (π) of 2.3 %, and *θ* (per site) of 0.01781. There were 33 nucleotide substitutions and one indel, from which we called 23 alleles.

The ITS2 data revealed strong partitioning by ploidy. Of the 23 alleles, three (H1, H20 and H21) were restricted to diploids, and 19 to tetraploids, with only one allele (H2) shared across ploidy levels (Table 1). H2 was not only shared across ploidy levels but was also the most widespread variant, found in 67 samples across 34 populations. This included geographically distinct species such as the Scottish endemic *E. marshallii* and the predominantly English and Welsh *E. anglica*, and ecologically contrasting taxa such as the dry heathland specialist *E. micrantha* and the (currently unpublished) obligate coastal ‘*E. fharaidensis*’. Overall, 86 % of taxa had one of six widespread alleles. There were also a large number of rare variants, with over two-thirds restricted to a single population (17 alleles: H4, H7–H10, H12–H21 and H23; Table 1). The remaining alleles found in multiple populations (H1, H2, H3, H5, H6, H11) showed no clear pattern of geography, with three found in all geographic regions (England, Scotland, Wales) and the remaining three shared between two geographic regions. Similarly, patterns of shared sequence variation do not follow species boundaries. Of the eight species with multiple populations (excluding hybrids), none of them had a diagnostic ITS2 sequence. Despite variants being shared across taxa, there was no evidence for this being due to non-neutral processes, as the value of Tajima’s *D* (−0.17) was not significantly different from zero.

**Table 1.** The distribution of ITS2 variation between species and geographic regions for British *Euphrasia*. Allele numbers correspond to the network in Fig. 4. Population-specific variants are aggregated under one column. *n* = number of samples. Regions refer to: W, Wales; S, Scotland; SW, South West England.

Taxa name	Region	*n*	Widespread haplotypes						Population-specific haplotypes
H1	H2	H3	H5	H6	H11
*E. anglica*	SW	4	4						0
*E. anglica*	W	3	2	1					0
*E. arctica*	S	3		1	2				0
*E. arctica*	SW	2		2					0
*E. arctica*	W	2		1	1				0
*E. arctica* × *confusa*	S	1							1
*E. arctica* × *foulaensis*	S	1		1					0
*E. arctica* × *micrantha*	S	3		1	1	1			0
*E. arctica* × *nemorosa*	S	2		1			1		0
*E. arctica* × *rostkoviana*	S	3		1	2				0
*E. cambrica*	W	3				1	1		1
*E. campbelliae*	S	3					3		0
*E. confusa*	S	4		4					0
*E. confusa*	SW	3			1	1			1
*E. confusa*	W	3		2					1
*E. confusa* × *micrantha*	S	2		1					1
“*E. fharaidensis”*	S	2		1				1	0
*E. foulaensis*	S	6		2	2		2		0
*E. foulaensis* × *marshllii*	S	2		1			1		0
*E. foulaensis* × *nemorosa*	S	1		1					0
*E. foulaensis* × *ostenfeldii*	S	1		1					0
*E. frigida*	S	6		5		1			0
*E. heslop-harrisonii*	S	6		5					1
*E. marshllii*	S	3		3					0
*E. marshallii* × *micrantha*	S	2		2					0
*E. micrantha*	S	5		4					1
*E. micrantha*	SW	3		2					1
*E. micrantha*	W	3						2	1
*E. micrantha* × *nemorosa*	SW	1			1				0
*E. micrantha* × *scottica*	W	4		4					0
*E. nemorosa*	S	3		3					0
*E. nemorosa*	SW	2		1					1
*E. nemorosa*	W	3		2					1
*E. nemorosa* × *tetraquetra*	SW	1		1					0
*E. ostenfeldii*	S	5		3					2
*E. ostenfeldii*	W	1		1					0
*E. pseudokerneri*	W	3			2		1		0
*E. rivularis*	W	3	2						1
*E. rostkoviana*	W	3	2						1
*E. rotundifolia*	S	1		1					0
*E. scottica*	S	3		3					0
*E. scottica*	W	3							3
*E. tetraquetra*	SW	3		3					0
*E. tetraquetra*	W	3		1			2		0
*E. tetraquetra* × *vigursii*	SW	2	1	1					0
*E. vigursii*	SW	4	4						0
Total		130	15	67	12	4	11	3	18

The putative hybrid species, *E. vigursii* and *E. rivularis*, possessed allele H1, which is common to other diploid taxa, or population-specific variants (H20, H21), but no tetraploid-specific allelic variation. The two sampled diploid–tetraploid hybrids (*E. arctica* × *rostkoviana*, *E. tetraquetra* × *vigursii*) possessed the full range of alleles: H2, which is common across ploidy levels, tetraploid-specific H3 and diploid-specific H1. Most (9/12) tetraploid hybrid populations had alleles shared with their putative parents, while the other populations had unique alleles.

The highest *F*_CT_ value in the SAMOVA was when *K* = 2 [see Supporting Information—Table S3], and this corresponded to the diploid–tetraploid divide described above. At *K* = 3, SAMOVA distinguished clusters corresponding to the two ploidy groups, and a third group of hybrid species derived from inter-ploidy mating. Analysis of molecular variance also supported the strong division by ploidy, with 88.2 % of variation attributed to ploidy differences (Table 2; *P* < 0.001). A high proportion of variation was also partitioned by taxa (63.2 %), and regions (25 %), though this may be inflated by limited sampling within species.

**Table 2.** Hierarchical AMOVA of British *Euphrasia* populations. Analyses performed between (A) taxa, (B) three geographic locations (Wales, South West England, Scotland), (C) diploids and tetraploids. Number in parentheses is the result only including species (excluding hybrids). d.f. = degrees of freedom. **P < 0.001; *P < 0.05.

Source of variation	ITS		Plastid DNA	
	d.f.	% Total variance	d.f.	% Total variance
(A) Taxa				
Between taxa	33 (19)	63.17** (65.62**)	33 (19)	15.87** (26.48**)
Within taxa	96 (77)	36.83 (34.38)	96 (68)	84.13 (73.52)
(B) Location				
Between regions	2 (2)	25.52** (25.92**)	2 (2)	5.40** (4.91*)
Within regions	127 (94)	74.48 (74.08)	127 (85)	94.60 (95.09)
(C) Ploidy				
Between ploidy groups	1 (1)	88.24** (84.39**)	1 (1)	11.05* (18.76*)
Within diploids and tetraploids	123 (95)	11.76 (15.61)	123 (86)	88.95 (81.24)

**Figure 4.**
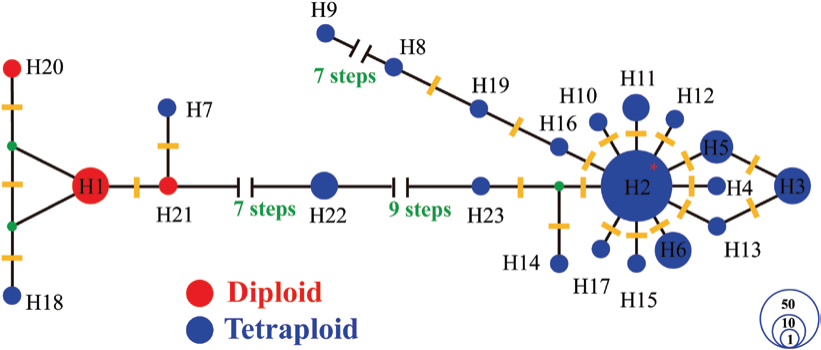
Median-joining network of ITS2 sequences in British *Euphrasia*. Numbers correspond to the ITS2 copies in Table 1. Alleles are coloured by ploidy, with diploids in red and tetraploids in blue. Hypothetical (unsampled) alleles are represented by filled green circles. H2 is the only allele shared across ploidy groups, found in 66 tetraploids and 1 diploid (indicate by the red asterisk). Yellow bar represents variation steps. The circle size represents the approximate numbers of individuals, and the scale is provided in the lower right corner.

Summary statistics and a network analysis revealed substantial divergence between diploid and tetraploid variants. An average of 18.3 site differences were found between unique diploid and tetraploid alleles, with divergence measured as *D*_XY_ = 0.051 (5.1 %). The phylogenetic network revealed broad clusters that largely corresponded to diploids and tetraploids, separated by many mutations (Fig. 4). The diploid cluster centres round allele H1, found in 15 individuals from five diploid species and one diploid–tetraploid hybrid. The only allele from this part of the network present in tetraploids is H18, found in a single sample of *E. ostenfeldii*. Within the predominantly tetraploid cluster, widespread variant H2 is at the centre (found across tetraploids and a single diploid individual), surrounded by other widespread tetraploid variants (H3, eight populations, 12 samples; H6, seven populations, 11 samples), and singleton variants.

#### Plastid haplotype diversity.

Initial sequencing of *rbcL* in 48 samples revealed no polymorphism and was excluded from subsequent analyses. The final *matK* alignment was 844 bp with one indel, and revealed 17 haplotypes. Our supplemental locus, the *rpl32-trnL* region, was 630 bp with nine indels. The final concatenated alignment of variable plastid loci (*matK* + *rpl32-trnL*) was 1474 bp for 130 samples, with 2.7 % segregating sites (40 sites) across 38 haplotypes. Nucleotide diversity was exceptionally low with π = 0.3 %, and *θ* (per site) was similarly low at 0.00381. Tajima’s *D* was not significantly different from zero (−0.27).

Similar to ITS, most plastid haplotypes were individual or population specific (63 %, 24/38 haplotypes found in one population only), with only four haplotypes being widespread (H4: 15 populations, 24 samples; H5: 13 populations, 18 samples; H2: 11 populations, 16 samples; H1, 10 populations, 14 samples; Supporting Information—Table S4). However, unlike ITS, plastid haplotypes revealed complex patterns unrelated to ploidy. Most widespread haplotypes were shared across ploidy levels. An AMOVA found a moderate degree of genetic diversity was partitioned by ploidy (18.7 %) and species (26.5 %), with these values being reduced when hybrids were included [see Supporting Information—Table S2]. Despite geography explaining little of the variation across the total data set (4.9 %), or being evident in the SAMOVA [see Supporting Information—Table S5], localized haplotype sharing was apparent in Scottish tetraploids. For example, haplotype H7 is shared across Scottish populations of *E. arctica* (and its hybrids), *E. foulaensis* and *E. micrantha* (and its hybrids), while H10 is also shared across three species in Scotland. Results from just the barcoding locus *mat*K were consistent with the concatenated alignment of variable plastid loci [see Supporting Information—Tables S6 and S7].

## Discussion

In this study, we used a broad-scale phylogenetic analysis and a regional DNA barcoding survey to investigate the evolution of taxonomically complex *Euphrasia* in Britain. Our phylogeny revealed deep divergence of ITS sequences between two clades of British *Euphrasia*, supporting the regional assembly of species diversity from a diverse pool of continental taxa. Our DNA barcoding survey revealed that British *Euphrasia* species do not possess diagnostic ITS and plastid sequence profiles, and instead variants are either widespread across taxa, or are individual or population specific. However, fixed sequence differences between sympatric diploids and tetraploids suggest ploidy is an important reproductive barrier partitioning genetic variation. Overall, our results shed light on the maintenance of genetic variation in one of the most renowned taxonomically challenging plant groups.

### Colonization history and polyploidy in British *Euphrasia*

Characterizing the factors driving species richness requires an understanding of the relative importance of local and regional processes. Our phylogenetic analysis allows us to estimate the number of times that *Euphrasia* has colonized Britain from mainland Europe, after which local speciation events give rise to endemic taxa. British taxa are found in two clades of the *Euphrasia* phylogeny, with each clade nested within diverse European relatives. The dated molecular phylogeny of Gussarova *et al.* (2008) shows divergence of the crown group including the two clades of British taxa occurred at around 8 million years ago (Ma), with diversity within each clade accumulating after that date but before c. 1 Ma (A. D. Twyford *et al.*, unpubl. res.). As such, clade divergence, and much of the speciation present within British *Euphrasia*, long pre-dates recent glacial divergence and the origin of young British endemic taxa. Overall, this suggests that British *Euphrasia* diversity has been assembled from a diverse pool of European and amphi-Atlantic taxa. Evidence for recurrent rounds of colonization from continental Europe have also been seen in phylogeographic studies of various other taxa, in particular tree species such as *Fagus sylvatica* (Rendell and Ennos 2003) and *Quercus petraea* (Cottrell *et al.* 2002).

It is notable from our DNA barcoding survey that ITS sequence variation is strongly partitioned by ploidy, despite the fact that diploids and tetraploids often grow in sympatry and are known to hybridize (Stace *et al.* 2015). This supports previous population surveys with AFLPs (French *et al.* 2008) where strong reproductive barriers are inferred between ploidy groups. The extent of divergence between diploid and tetraploid ITS sequences adds further weight to the British tetraploids being allopolyploids. Other lines of support for an allotetraploid origin come from the high number of tetraploid-specific AFLP bands (French *et al.* 2008), fixed microsatellite heterozygosity indicative of disomic inheritance (Stone 2013), and tetraploid genome assemblies of double the size of the diploids (A. D. Twyford and R. W. Ness, unpubl. data). While it is difficult to decipher the parentage of British tetraploids from our data, our phylogenetic analysis places these tetraploid British taxa in a clade composed exclusively of tetraploids (Clade IVd, Gusarova *et al.* 2008), and thus the polyploid event likely pre-dates dispersal to the UK.

### DNA barcoding in taxonomically complex genera

Taxonomically complex *Euphrasia* have many characteristics that would make a DNA-based identification system desirable. In particular, molecular identification tools could be used to confirm species identities and subsequently revise the distribution of these under-recorded taxa. More generally, genetic data could be used to investigate which British species are genetically cohesive ‘good’ taxa. Our data show, however, that barcode sequences do not correlate with species boundaries as defined by morphology. Instead, variants are often individual or population specific (for both ITS and plastid DNA), and where widespread are either shared across species within a ploidy level (for ITS) or across ploidy levels and species (plastid DNA). Patterns of shared sequences are often surprising, including between geographically disparate populations 100+ km apart, and between ecologically specialized taxa that seldom co-occur in the wild.

One possibility is that the species are not discrete genetic entities, and that the current taxonomy reflects a blend of discrete lineages, polytopic taxa and morphotypes determined by a small number of genes. Alternatively, the species may represent meaningful biological entities, but with boundaries permeable to gene flow. In either case, the lack of species-specific or morphotype-specific barcodes may be attributed to one of many evolutionary factors, including recent speciation, hybridization and self-fertilization. Recent speciation and lack of sequence variation no doubt affect the resolution of our phylogeny, and in part affect our DNA barcoding survey to look at patterns of shared haplotypic variation. While elevated plastid diversity is a hallmark of some parasitic taxa, this is not the case for facultative hemiparasites like *Euphrasia*, which generally show similar patterns of mutation to autotrophic taxa (Wicke *et al.* 2016). The opposing forces of self-fertilization, which results in population-level differentiation, and hybridization, which may cause transpecific polymorphism, will leave a complex signal of genetic variation. The large number of reported hybrids and hybrid species based on morphology (Preston and Pearman 2015; Stace *et al.* 2015), as well as the prevalence of hybridization in genetic data (Liebst 2008; Stone 2013), point to hybridization being a key factor shaping genetic diversity in *Euphrasia*. Future genomic surveys will pinpoint the scale at which genetic variation is partitioned, and estimate the proportion of loci introgressing across species barrier in models that explicitly account for incomplete lineage sorting (Twyford and Ennos 2012).

The sharing of DNA barcode sequences among related *Euphrasia* species parallels a number of other studies where DNA barcoding has failed to provide species-level information. Extensive plastid haplotype sharing has been reported between two species of *Rhinanthus* (Vrancken *et al.* 2012), a related genus in the Orobanchaceae. Another notable example is in willows, where hybridization and selective sweeps have caused a single haplotype to spread across highly divergent taxa and between geographic regions (Percy *et al.* 2014; Twyford, 2014). Poor species discrimination from DNA barcoding is also seen in the rapid radiation of Chinese *Primula* (Yan *et al.* 2015a) and *Rhododendron* (Yan *et al.* 2015b), which is likely a product of hybridization and recent species divergence.

Future DNA barcoding systems that target large quantities of nuclear sequence variation are extremely promosing for species discrimination in taxonomically complex groups (Coissac *et al.* 2016; Hollingsworth *et al.* 2016). These data have the joint benefit of providing many nucleotide characters from unlinked loci, while also moving away from genomic regions that have atypical inheritance and patterns of evolution (i.e. plastids). Analyses of many nuclear genes (or entire genomes) would be particularly valuable for *Euphrasia*, where it may be possible to identify adaptive variants maintained in the face of hybridization. These adaptive genes may underlie differences between species or ecotypes (Twyford and Friedman 2015), and these loci could then potentially be used for future species identification.

## Conclusions

This study highlights how DNA barcoding data may fail to distinguish between species in taxonomically complex groups such as *Euphrasia*. No species in our study possessed a consistent diagnostic sequence profile. Widespread haplotype sharing among species, in conjunction with high levels of intraspecific variation, makes *Euphrasia* a particular challenge for DNA barcoding. However, our results are able to help us understand the maintenance of genetic diversity and the evolutionary importance of polyploidy.

## Accession Numbers

All sequences obtained from fresh plant specimens have been deposited in the GenBank database under accession numbers MH202267–MH202656.

## Sources of Funding

This study formed part of an international exchange programme for X.W., funded by the Chinese Scholarship Council (CSC). P.M.H. and M.R. acknowledge funding from the Scottish Government’s Rural and Environment Science and Analytical Services Division (RESAS). Research by G.G. is supported by the Research Council of Norway: Projects N248799 and N257642; and Norwegian Taxonomy Initiative: Project N70184215. Research by A.D.T. is supported by a Natural Environment Research Council (NERC) Fellowship NE/L011336/1.

## Contributions by the Authors

A.D.T and P.M.H. conceived and designed the study. A.D.T., G.G., N.d.V. and C.M. provided samples. C.M. identified the specimens. X.W. and M.R. performed the sequencing. X.W., G.G., M.R. and A.D.T analysed the data. A.D.T. and X.W. wrote the manuscript, with comments from all other authors.

## Conflict of Interest

None declared.

## Supporting Information

The following additional information is available in the online version of this article—

**Table S1.** Voucher information and population details of British *Euphrasia* samples used for UK DNA barcoding. The cpDNA regions include *matK* and *rpl32-trnL*, with *rbcL* generated for a subset of taxa but excluded from the final analysis (see main text). The columns headed ‘cpDNA’ and ‘ITS 2’ indicate whether the sample was successfully sequenced (1) or not (0). Taxa with an asterisk were also included in the global phylogenetic analysis.

**Table S2.** PCR conditions and primer sequences for regions sequenced in this study. Loci used for DNA barcoding were *matK*, *rbcL* and *ITS2*, supplemented with *rpl32-trnL.* Regions used for phylogenetic analysis were *atpB-rbcL*, *trnL* intron, *trnL-trnF* and *ITS*.

**Table S3.** Spatial analysis of molecular variation (SAMOVA) of ITS sequence data across British *Euphrasia* populations.

**Table S4.** Plastid haplotype frequencies across British *Euphrasia* species.

**Table S5.** Spatial analysis of molecular variation (SAMOVA) of plastid sequence data across British *Euphrasia* populations.

**Table S6.** The distribution of matK haplotypes for populations of British *Euphrasia*. The cpDNA (*n*) column indicates the sample sizes.

**Table S7.** Hierarchical analysis of molecular variance (AMOVA) for *matK* sequenced in British *Euphrasia* populations.

## Supplementary Material

Supplementary Tables
