# Supplementary material for: DNA barcoding a taxonomically complex hemiparasitic genus reveals deep divergence between ploidy levels but lack of species-level resolution
Source: AoB Plants. 2018 Apr 24;10(3):ply026. doi: 10.1093/aobpla/ply026 (PMC5941139; doi:10.1093/aobpla/ply026)
Supplement: Supplementary Tables [file ply026_suppl_supplementary_tables.doc]

Supplementary Table S1. Voucher information and population details of British *Euphrasia* samples used for UK DNA barcoding. The cpDNA regions include *matK* and *rpl32-trnL*, with *rbcL* generated for a subset of taxa but excluded from the final analysis (see main text)*.* The columns headed ‘cpDNA’ and ‘ITS 2’ indicate whether the sample was successfully sequenced (1) or not (0). Taxa with an asterisk were also included in the global phylogenetic analysis.

| **Taxa** | **Sample ID** | **Ploidy** | **cp DNA** | **ITS 2** | **Region** | **Latitude** | **Longitude** | **Locality** | **Collector(s)** |
| --- | --- | --- | --- | --- | --- | --- | --- | --- | --- |
| *E. anglica* | NMW2621 | 2x | 1 | 1 | Wales | 51.588311 | -3.311379 | Dyffryn, Glamorgan | A.E. Wade |
| *E. anglica** | NMW332 | 2x | 1 | 1 | Wales | 51.68899918 | -3.108069897 | Cefn-crib, Monmouthshire | T.G. Evans |
| *E. anglica* | NMW2620 | 2x | 1 | 1 | Wales | 52.30537735 | -4.022516611 | Mynydd Bach, Cardiganshire | A.O. Chater |
| *E. anglica** | SCP | 2x | 1 | 1 | SW England | 50.911983 | -1.647611 | Stony Cross Plain, Hampshire | A. Twyford |
| *E. anglica* | JAP | 2x | 1 | 1 | SW England | 50.921778 | -1.650528 | Janesmoor Pond, Hampshire | A. Twyford |
| *E. anglica* | BED | 2x | 1 | 1 | SW England | 50.5135 | -4.113306 | Bedford Bridge, Devon | A. Twyford |
| *E. anglica* | SHC | 2x | 1 | 1 | SW England | 50.623917 | -3.846278 | Shapley Common | A. Twyford |
| *E. arctica** | NMW2623 | 4x | 1 | 1 | Wales | 51.57888813 | -3.675454646 | Llangynwyd, Glamorgan | A.E. Wade; R.G. Ellis |
| *E. arctica* | NMW334 | 4x | 1 | 1 | Wales | 52.05189896 | -3.825089931 | Penstacan, Carmarthenshire | J. Iliff |
| *E. arctica** | NSF | 4x | 1 | 1 | SW England | 50.532083 | -4.522139 | Nine Stone Farm, Cornwall | A. Twyford |
| *E. arctica* | CRR | 4x | 1 | 1 | SW England | 50.623861 | -4.628667 | Crowdy Reservoir, Cornwall | A. Twyford |
| *E. arctica** | 113 | 4x | 1 | 1 | Scotland | 59.248 | -2.583 | Sanday, Orkney islands | C. Metherell; H. Metherell;  F. Rumsey |
| *E. arctica* | 135 | 4x | 1 | 1 | Scotland | 59.136644 | -3.3141345 | Geo, Orkney islands | C. Metherell; H. Metherell;  F. Rumsey |
| *E. arctica* | 159 | 4x | 1 | 1 | Scotland | 58.506 | -4.384 | Coldbackie, Sutherland | C. Metherell; H. Metherell;  F. Rumsey |
| *E. arctica* x *confusa* | 132 | 4x | 1 | 1 | Scotland | 59.100137 | -3.35324 | Marwick Head, Orkney islands | C. Metherell; H. Metherell;  F. Rumsey |
| *E. arctica* x *foulaensis* | 139 | 4x | 1 | 1 | Scotland | 58.616009 | -3.4689336 | West Murkle, Caithness | C. Metherell; H. Metherell;  F. Rumsey |
| *E. arctica* x *micrantha* | 117 | 4x | 1 | 1 | Scotland | 58.923266 | -3.3296379 | Hoy, Orkney islands | C. Metherell; H. Metherell;  F. Rumsey |
| *E. arctica* x *micrantha* | 119 | 4x | 1 | 1 | Scotland | 58.921898 | -3.3316695 | Hoy, Orkney islands | C. Metherell; H. Metherell;  F. Rumsey |
| *E. arctica* x *micrantha* | 122 | 4x | 1 | 1 | Scotland | 58.919203 | -3.3404227 | Hoy, Orkney islands | C. Metherell; H. Metherell;  F. Rumsey |
| *E. arctica* x *nemorosa* | 129 | 4x | 1 | 1 | Scotland | 58.893417 | -2.7062147 | Deerness, Orkney islands | C. Metherell; H. Metherell;  F. Rumsey |
| *E. arctica* x *nemorosa* | 160 | 4x | 1 | 1 | Scotland | 59.424176 | -2.6871063 | Coldbackie, Caithness | C. Metherell; H. Metherell;  F. Rumsey |
| *E. arctica* x *rostkoviana* | 146 | 4x | 1 | 1 | Scotland | 58.562989 | -3.7895616 | Reay, Caithness | C. Metherell; H. Metherell;  F. Rumsey |
| *E. arctica* x *rostkoviana* | 147 | 4x | 1 | 1 | Scotland | 58.473225 | -3.7848238 | Reay, Caithness | C. Metherell; H. Metherell;  F. Rumsey |
| *E. arctica* x *rostkoviana* | 148 | 4x | 1 | 1 | Scotland | 58.562538 | -3.7897105 | Reay, Caithness | C. Metherell; H. Metherell;  F. Rumsey |
| *E. cambrica** | NMW2624 | 4x | 1 | 1 | Wales | 53.08160019 | -4.083360195 | Clogwyn Coch, Caernarvonshire | A.J. Silverside; T.C.G. Rich;  B. Jones; A. Jones |
| *E. cambrica* | NMW335 | 4x | 1 | 1 | Wales | 53.08160019 | -4.083360195 | Clogwyn Coch, Caernarvonshire | A.J. Silverside; T.C.G. Rich;  B. Jones; A. Jones |
| *E. cambrica* | 434 | 4x | 1 | 1 | Wales | 53.083 | -4.083 | Snowdon, Caernarvanshire | G. French |
| *E. campbelliae** | RBGE301 | 4x | 1 | 1 | Scotland | 58.09686441 | -7.104816888 | Mealasta, Outer Hebrides | C. Metherell |
| *E. campbelliae* | RBGE303 | 4x | 1 | 1 | Scotland | 58.10385028 | -7.107857344 | Mealasta, Outer Hebrides | C. Metherell |
| *E. campbelliae* | RBGE304 | 4x | 1 | 1 | Scotland | 58.13798851 | -7.111396151 | Islibhig, Outer Hebrides | C. Metherell |
| *E. confusa* | NMW337 | 4x | 1 | 1 | Wales | 51.64599991 | -3.699609995 | Cynonville, Glamorgan | A.E. Wade |
| *E. confusa* | NMW2626 | 4x | 1 | 1 | Wales | 51.71634279 | -4.367170869 | Tywyn Burrows, Carmarthenshire | R.D. Pryce; K.A. Pryce |
| *E. confusa** | NMW2625 | 4x | 1 | 1 | Wales | 52.51932763 | -3.917364288 | Ystrad Einion, Cardiganshire | A.O. Chater |
| *E. confusa* | WHP | 4x | 1 | 1 | SW England | 50.811333 | -1.715972 | Whitten Pond, Hampshire | A. Twyford |
| *E. confusa** | SHA | 4x | 1 | 1 | SW England | 50.557833 | -4.073389 | Sharpitor, Devon | A. Twyford |
| *E. confusa* | 284 | 4x | 1 | 1 | SW England | 50.469 | -4.65 | Bunny Hill, East Cornwall | G. French |
| *E. confusa* | 1349 | 4x | 1 | 1 | Scotland | 58.135 | -4.935 | Inchadamp, West Sutherland | G. French |
| *E. confusa* | 1350 | 4x | 1 | 1 | Scotland | 58.135 | -4.935 | Inchadamp, West Sutherland | G. French |
| *E. confusa* | 1351 | 4x | 1 | 1 | Scotland | 58.135 | -4.935 | Inchadamp, West Sutherland | G. French |
| *E. confusa** | 145 | 4x | 1 | 1 | Scotland | 58.623 | -3.536 | Holborn Head, Caithness, | C. Metherell; H. Metherell;  F. Rumsey |
| *E. confusa* x *micrantha* | 141 | 4x | 1 | 1 | Scotland | 58.623 | -3.536 | Holborn Head, Caithness | C. Metherell; H. Metherell;  F. Rumsey |
| *E. confusa* x *micrantha* | 169 | 4x | 1 | 1 | Scotland | 58.53599 | -4.4649116 | Altnaharra, Caithness | C. Metherell; H. Metherell;  F. Rumsey |
| *‘E. fharaidensis’* | 130 | 4x | 1 | 0 | Scotland | 58.964532 | -2.7085383 | Deerness, Orkney islands | C. Metherell; H. Metherell;  F. Rumsey |
| *‘E. fharaidensis’** | 156 | 4x | 1 | 1 | Scotland | 58.518249 | -4.2622308 | Brogie Estuary, Torrisdale Bay | C. Metherell; H. Metherell;  F. Rumsey |
| *‘E. fharaidensis’* | 167 | 4x | 1 | 1 | Scotland | 58.53599 | -4.4649116 | Fahraid Head, Caithness |  |
| *E. foulaensis** | RBGE302 | 4x | 1 | 1 | Scotland | 58.09667315 | -7.107338435 | Mealasta, Outer Hebrides | C. Metherell |
| *E. foulaensis* | RBGE305 | 4x | 1 | 1 | Scotland | 58.18546435 | -7.094207187 | Mangersta, Outer Hebrides | C. Metherell |
| *E. foulaensis* | RBGE306 | 4x | 1 | 1 | Scotland | 58.51231678 | -6.254114237 | Eropaid, Outer Hebrides | C. Metherell |
| *E. foulaensis* | 125 | 4x | 1 | 1 | Scotland | 58.960231 | -2.7068853 | Deerness, Orkney islands | C. Metherell; H. Metherell;  F. Rumsey |
| *E. foulaensis* | 126 | 4x | 1 | 1 | Scotland | 58.960321 | -2.7067133 | Deerness, Orkney islands | C. Metherell; H. Metherell;  F. Rumsey |
| *E. foulaensis* | 138 | 4x | 1 | 1 | Scotland | 58.609707 | -3.4756923 | West Murkle, Caithness | C. Metherell; H. Metherell;  F. Rumsey |
| *E. foulaensis* x *marshallii* | 100 | 4x | 1 | 1 | Scotland | 59.021857 | -3.3581759 | Yesnaby, Orkney islands | C. Metherell; H. Metherell;  F. Rumsey |
| *E. foulaensis* x *marshallii* | 131 | 4x | 1 | 1 | Scotland | 59.279689 | -3.36036 | Marwick Head, Orkney islands | C. Metherell; H. Metherell;  F. Rumsey |
| *E. foulaensis* x *nemorosa* | 163 | 4x | 1 | 1 | Scotland | 58.482511 | -4.6721871 | An t'Sron, Caithness | C. Metherell; H. Metherell;  F. Rumsey |
| *E. foulaensis* x *ostenfeldii* | 144 | 4x | 1 | 1 | Scotland | 58.623 | -3.536 | Holborn Head, Caithness | C. Metherell; H. Metherell;  F. Rumsey |
| *E. frigida* | NMW7712 | 4x | 1 | 1 | Scotland | 56.467396 | -4.319724 | Craig an Lochan, Perthshire, Mid | F. Rose |
| *E. frigida* | NMW7714 | 4x | 0 | 1 | Scotland | 57.08279284 | -3.639703422 | Coire Odhar, Easterness | F. Rose |
| *E. frigida** | NMW7713 | 4x | 1 | 1 | Scotland | 60.14447605 | -2.091836881 | Soberlie Hill, Shetland | W.A. Scott |
| *E. frigida* | 1802 | 4x | 1 | 1 | Scotland | 56.541 | -4.29 | Creag an Lochain, Mid Perth | G. French |
| *E. frigida* | 1803 | 4x | 1 | 1 | Scotland | 56.541 | -4.29 | Creag an Lochain, Mid Perth | G. French |
| *E. frigida* | 1807 | 4x | 1 | 1 | Scotland | 56.541 | -4.29 | Creag an Lochain, Mid Perth | G. French |
| *E. heslop-harrisonii* | RBGE307 | 4x | 1 | 1 | Scotland | 57.8102904 | -7.060114311 | Northton, Orkney | C. Metherell |
| *E. heslop-harrisonii* | NMW7715 | 4x | 0 | 1 | Scotland | 58.55 | -4 | Strathy, Sutherland, West | F. Rose |
| *E. heslop-harrisonii** | NMW7716 | 4x | 1 | 1 | Scotland | 60.33188391 | -1.440418437 | Holms of Uyea-sound, Shetland | W.A. Scott |
| *E. heslop-harrisonii* | 107 | 4x | 1 | 1 | Scotland | 59.287134 | -2.4387893 | Sanday, Orkney islands | C. Metherell; H. Metherell;  F. Rumsey |
| *E. heslop-harrisonii* | 110 | 4x | 1 | 1 | Scotland | 59.285163 | -2.4879062 | Sanday, Orkney islands | C. Metherell; H. Metherell;  F. Rumsey |
| *E. heslop-harrisonii* | 127 | 4x | 1 | 0 | Scotland | 58.962839 | -2.7060694 | Deerness, Orkney islands | C. Metherell; H. Metherell;  F. Rumsey |
| *E. heslop-harrisonii* | 128 | 4x | 1 | 1 | Scotland | 58.963477 | -2.7045175 | Deerness, Orkney islands | C. Metherell; H. Metherell;  F. Rumsey |
| *E. marshallii** | 151 | 4x | 1 | 1 | Scotland | 58.571938 | -3.9344687 | Rubh Bhra, Caithness, | C. Metherell; H. Metherell;  F. Rumsey |
| *E. marshallii* | 1616 | 4x | 1 | 1 | Scotland | 58.534 | -4.218 | Betty Hill, West Sutherland | G. French |
| *E. marshallii* | 1621 | 4x | 1 | 1 | Scotland | 58.534 | -4.218 | Betty Hill, West Sutherland | G. French |
| *E. marshallii* x *micrantha* | 149 | 4x | 1 | 1 | Scotland | 58.570692 | -3.9337121 | Rubh Bhra, Caithness | C. Metherell; H. Metherell;  F. Rumsey |
| *E. marshallii* x *micrantha* | 152 | 4x | 1 | 1 | Scotland | 58.571146 | -3.9333932 | Rubh Bhra, Caithness | C. Metherell; H. Metherell;  F. Rumsey |
| *E. micrantha** | NMW2628 | 4x | 1 | 1 | Wales | 52.12535824 | -3.812749224 | Afon Doethe, Cardiganshire | A.O. Chater; J.P. Woodman |
| *E. micrantha* | NMW2627 | 4x | 1 | 1 | Wales | 52.20711777 | -3.757534197 | Nantystalwyn, Cardiganshire | A.O. Chater; R.D. Pryce |
| *E. micrantha* | NMW336 | 4x | 1 | 1 | Wales | 52.42279816 | -3.910959959 | Cwmerfyn, Cardiganshire | A.O. Chater |
| *E. micrantha** | STC | 4x | 1 | 1 | SW England | 50.907423 | -1.620662 | Malwood near Stony Cross, Hampshire | A. Twyford |
| *E. micrantha* | 180 | 4x | 1 | 1 | SW England | 50.303 | -5.234 | Chapel Porth, West Cornwall | G. French |
| *E. micrantha* | 184 | 4x | 1 | 1 | SW England | 50.303 | -5.234 | Chapel Porth, West Cornwall | G. French |
| *E. micrantha* | 104 | 4x | 1 | 1 | Scotland | 59.02026 | -3.3389535 | Yesnaby, Orkney islands | C. Metherell; H. Metherell;  F. Rumsey |
| *E. micrantha* | 120 | 4x | 1 | 1 | Scotland | 58.92237 | -3.338114 | Hoy, Orkney islands | C. Metherell; H. Metherell;  F. Rumsey |
| *E. micrantha* | 142 | 4x | 1 | 1 | Scotland | 58.623 | -3.536 | Holborn Head, Caithness | C. Metherell; H. Metherell;  F. Rumsey |
| *E. micrantha* | 154 | 4x | 1 | 1 | Scotland | 58.541668 | -4.2993104 | Skerray, Caithness | C. Metherell; H. Metherell;  F. Rumsey |
| *E. micrantha** | 157 | 4x | 1 | 1 | Scotland | 58.738326 | -4.3046777 | Borgie Bridge | C. Metherell; H. Metherell;  F. Rumsey |
| *E. micrantha* x *nemorosa* | WAR | 4x | 1 | 1 | SW England | 50.484389 | -4.627444 | Warleggan, Cornwall | A. Twyford |
| *E. micrantha* x *scottica* | 103 | 4x | 1 | 1 | Scotland | 59.020086 | -3.3384242 | Yesnaby, Orkney islands | C. Metherell; H. Metherell;  F. Rumsey |
| *E. micrantha* x *scottica* | 118 | 4x | 1 | 0 | Scotland | 58.923532 | -3.3299955 | Hoy, Orkney islands | C. Metherell; H. Metherell;  F. Rumsey |
| *E. micrantha* x *scottica* | 121 | 4x | 1 | 1 | Scotland | 58.92237 | -3.338114 | Hoy, Orkney islands | C. Metherell; H. Metherell;  F. Rumsey |
| *E. micrantha* x *scottica* | 123 | 4x | 1 | 1 | Scotland | 58.870528 | -3.2502675 | Hoy, Orkney islands | C. Metherell; H. Metherell;  F. Rumsey |
| *E. micrantha* x *scottica* | 164 | 4x | 1 | 1 | Scotland | 58.482752 | -4.6735779 | Ant'Sron, Caithness | C. Metherell; H. Metherell;  F. Rumsey |
| *E. nemorosa** | NMW2630 | 4x | 1 | 1 | Wales | 51.389811 | -3.345153 | Rhoose, Glamorgan | A.E. Wade |
| *E. nemorosa* | NMW339 | 4x | 1 | 1 | Wales | 51.62310028 | -2.738820076 | Caerwent, Monmouthshire | T.G. Evans |
| *E. nemorosa* | NMW2629 | 4x | 1 | 1 | Wales | 51.76843141 | -2.725978618 | Trelleck Hill, Monmouthshire | T.G. Evans |
| *E. nemorosa* | CHC | 4x | 1 | 1 | SW England | 50.971104 | -0.968344 | Chapel Common, Hampshire | A. Twyford |
| *E. nemorosa** | BUN | 4x | 1 | 1 | SW of England | - | - |  | A. Twyford |
| *E. nemorosa* | 1676 | 4x | 1 | 1 | Scotland | 58.517 | -4.219 | Betty Hill, West Sutherland | G. French |
| *E. nemorosa** | 1678 | 4x | 1 | 1 | Scotland | 58.517 | -4.219 | Betty Hill, West Sutherland | G. French |
| *E. nemorosa* | 1679 | 4x | 1 | 1 | Scotland | 58.517 | -4.219 | Betty Hill, West Sutherland | G. French |
| *E. nemorosa* x *tetraquetra* | NAN | 4x | 1 | 1 | SW of England | 50.1065 | -5.697472 | Nanjulian Cliff, Cornwall | A. Twyford |
| *E. ostenfeldii** | NMW340 | 4x | 1 | 1 | Wales | 53.07979965 | -4.084770203 | Clogwyn Coch, Caernarvonshire | A.J. Silverside; T.C.G. Rich;  B. Jones; A. Jones |
| *E. ostenfeldii** | NMW2631 | 4x | 1 | 1 | Scotland | 59.94540834 | -1.357446863 | Colsay, Shetland | W.A. Scott |
| *E. ostenfeldii* | NMW2632 | 4x | 1 | 1 | Scotland | 60.408159 | -1.430034 | Egilsay, Shetland | W.A. Scott |
| *E. ostenfeldii* | 134 | 4x | 1 | 1 | Scotland | 59.137376 | -3.3127644 | Geo, Orkney islands | C. Metherell; H. Metherell;  F. Rumsey |
| *E. ostenfeldii* | 137 | 4x | 1 | 1 | Scotland | 59.138156 | -3.3120427 | Geo, Orkney islands | C. Metherell; H. Metherell;  F. Rumsey |
| *E. ostenfeldii* | 162 | 4x | 1 | 1 | Scotland | 58.482443 | -4.6711525 | Ant'Sron, Caithness | C. Metherell; H. Metherell;  F. Rumsey |
| *E. pseudokerneri** | NMW338 | 4x | 1 | 1 | Wales | 51.61989975 | -2.759720087 | Dinham, Monmouthshire | T.G. Evans |
| *E. pseudokerneri* | NMW2634 | 4x | 1 | 1 | Wales | 52.10908824 | -4.628471398 | Penparc, Cardiganshire | A.O. Chater |
| *E. pseudokerneri* | NMW2633 | 4x | 1 | 1 | Wales | 52.3817234 | -3.805602982 | Mynach Vale, Cardiganshire | A.O. Chater; P.A. Smith |
| *E. rivularis** | NMW2636 | 2x | 1 | 1 | Wales | 51.89886246 | -3.746928685 | Llanddeusant, Carmarthenshire | S. Thomas; J. Iliff |
| *E. rivularis* | NMW2635 | 2x | 1 | 1 | Wales | 53.02890015 | -4.089029789 | Snowdon, Caernarvonshire | T.C.G. Rich |
| *E. rivularis* | NMW333 | 2x | 1 | 1 | Wales | 53.07979965 | -4.084770203 | Clogwyn Coch, Caernarvonshire | A.J. Silverside; T.C.G. Rich;  B. Jones; A. Jones |
| *E. rostkoviana* | NMW2638 | 2x | 0 | 1 | Wales | 51.76292483 | -3.75761531 | Ystalyfera, Glamorgan | Q.O.N. Kay |
| *E. rostkoviana** | NMW341 | 2x | 1 | 1 | Wales | 52.36000061 | -3.845099926 | Strata Florida, Cardiganshire | A.O. Chater; J.P. Woodman |
| *E. rostkoviana* | NMW2639 | 2x | 1 | 1 | Wales | 52.739618 | -3.828735 | Aeron Fechan stream, Cardiganshire | A.O. Chater |
| *E. rotundifolia* * | 153 | 4x | 1 | 1 | Scotland | 58.572786 | -3.9383849 | Rubh Bhra, Caithness | C. Metherell; H. Metherell;  F. Rumsey |
| *E. scottica* | NMW2641 | 4x | 1 | 1 | Wales | 52.13564291 | -3.845313536 | Afon Pysgotwr Fach, Cardiganshire | A.O. Chater; P.A. Smith |
| *E. scottica** | NMW343 | 4x | 1 | 1 | Wales | 52.32040024 | -3.847860098 | Ysbyty Ystwyth, Cardiganshire | A.O. Chater |
| *E. scottica* | NMW2640 | 4x | 1 | 1 | Wales | 52.381599 | -3.922482 | Rhydtalog, Cardiganshire | A.O. Chater |
| *E. scottica* | 1812 | 4x | 1 | 1 | Scotland | 56.54 | -4.288 | Creag an Lochain, Mid Perth | G. French |
| *E. scottica* | 1814 | 4x | 1 | 1 | Scotland | 56.54 | -4.288 | Creag an Lochain, Mid Perth | G. French |
| *E. scottica** | 1816 | 4x | 1 | 1 | Scotland | 56.54 | -4.288 | Creag an Lochain, Mid Perth | G. French |
| *E. tetraquetra* | NMW2643 | 4x | 1 | 1 | Wales | 51.53760017 | -4.213242048 | Port Eynon, Glamorgan | G. Hutchinson |
| *E. tetraquetra** | NMW342 | 4x | 1 | 1 | Wales | 52.13800049 | -4.644050121 | Foel-y-mwnt, Cardiganshire | A.O. Chater |
| *E. tetraquetra* | NMW2642 | 4x | 1 | 1 | Wales | 52.19113544 | -4.407978478 | Cwmtydu, Cardiganshire | A.O. Chater |
| *E. tetraquetra** | KAV | 4x | 1 | 1 | SW of England | 50.241722 | -5.380472 | Knavock, Cornwall | A. Twyford |
| *E. tetraquetra* | 96 | 4x | 1 | 1 | SW of England | 50.283 | -5.236 | Porthtowan, West Cornwall | G. French |
| *E. tetraquetra* | 99 | 4x | 1 | 1 | SW of England | 50.283 | -5.236 | Porthtowan, West Cornwall | G. French |
| *E. tetraquetra* x *vigursii* | CAR | 4x | 1 | 1 | SW of England | 50.258778 | -5.303972 | Carvannel, Cornwall | A. Twyford |
| *E. tetraquetra* x *vigursii* | PHI | 4x | 1 | 1 | SW of England | 50.201806 | -5.41075 | Phillack Towans, Cornwall | A. Twyford |
| *E. vigursii* | NMW7711 | 2x | 1 | 1 | SW of England | 50.48475999 | -4.630293135 | Treslea Downs, Cornwall, East | D.T. Holyoak |
| *E. vigursii** | BUH | 2x | 1 | 1 | SW of England | 50.478278 | -4.650861 | Bunny's Hill, Cornwall | A. Twyford |
| *E. vigursii* | 101 | 2x | 1 | 1 | SW of England | 50.251 | -5.301 | Carvannel Downs, West Cornwall | G. French |
| *E. vigursii* | 105 | 2x | 1 | 1 | SW of England | 50.251 | -5.301 | Carvannel Downs, West Cornwall | G. French |

Supplementary Table S2. PCR conditions and primer sequences for regions sequenced in this study. Loci used for DNA barcoding were *matK*, *rbcL* and *ITS2*, supplemented with *rpl32-trnL. Regions used for phylogenetic analysis were atpB-rbcL*, *trnL* intron, *trnL-trnF* and *ITS*.

| Region | Primer | Orient  -ation | Sequence (5'-3' ) | DNA amplifications | PCR conditions | References |
| --- | --- | --- | --- | --- | --- | --- |
| *atpB-rbcL* | atpB-rbcL. F | F | ACATCKARTACKGGACCAATAA | 1×PCR buffer, 0.05 U Taq polymerase, 0.6 mM MgCl_2_, 1.0 mM of each dNTP, 0.25 mM of each primer and 1 μL of template DNA | 5 min at 94 ^o^C, 30× (30 s at 94 ^o^C, 30 s at 54 ^o^C, 2 min at 72 ^o^C), 10 min at 72 ^o^C | Hodges and Arnold, 1994 |
|  | atpB-rbcL. R | R | AACACCAGCTTTRAATCCAA |  |  |  |
| ITS | ITS 4 | F | TCCTCCGCTTATTGATATGC | 1×PCR buffer, 0.05 U Taq polymerase, 0.6 mM MgCl_2_, 1.0 mM of each dNTP, 0.25 mM of each primer and 1 μL of template DNA | 5 min at 94 ^o^C, 30× (30 s at 94 ^o^C, 30 s at 54 ^o^C, 2 min at 72 ^o^C), 10 min at 72 ^o^C | White et al. 1990 |
|  | ITS 5 | R | GGAAGTAAAAGTCGTAACAAGG |  |  |  |
| ITS2 | S2F | F | ATGCGATACTTGGTGTGAAT | 1×PCR buffer, 0.05 U Taq polymerase, 0.4 mM MgCl_2_, 1.0 mM of each dNTP, 0.25 mM of each primer and 1 μL of template DNA | 5 min at 94 ^o^C, 40× (30 s at 94 ^o^C, 30 s at 50 ^o^C, 45 s at 72 ^o^C), 10 min at 72 ^o^C | Chen et al. 2010 |
|  | S3R | R | ATTGTAGTCTGGAGAAGCGTC |  |  |  |
| *matK* | trnK-3914F | F | ATCTGGGTTGCTAACTCAATGG | 1×PCR buffer, 0.05 U Taq polymerase, 0.4 mM MgCl_2_, 1.0 mM of each dNTP, 0.25 mM of each primer and 1 μL of template DNA | 5 min at 94 ^o^C, 40× (30 s at 94 ^o^C, 60 s at 50 ^o^C, 45 s at 72 ^o^C), 10 min at 72 ^o^C | Whitten et al. 2000 |
|  | matK-1520R | R | CGGATAATGTCCAAATAC CAAATA |  |  |  |
| *rbcL* | rbcL aaF | F | ATGTCACCACAAACAGAGACTAAAGC | 1×PCR buffer, 0.05 U Taq polymerase, 2.5 mM MgCl_2_, 0.2 mM of each dNTP, 0.20 mM of each primer and 1 μL of template DNA | 2 min at 94 ^o^C, 35x (1 min at 94 ^o^C, 1 min at 45 ^o^C, 2 min at 72 ^o^C), 10 min at 72 ^o^C | Kress and Erickson, 2007 |
|  | rbcL aaR | R | CTTCTGCTACAAATAAGAATCGATCTC |  |  |  |
| *rpl32-trnL^UAG^* | rpL32-F | F | CAGTTCCAAAAAAACGTACTTC | 1×PCR buffer, 0.05 U Taq polymerase, 0.6 mM MgCl_2_, 1.0 mM of each dNTP, 0.25 mM of each primer and 1 μL of template DNA | 5 min at 94 ^o^C, 35× (30 s at 94 ^o^C, 45 s at 50 ^o^C, 40 s at 72 ^o^C), 5 min at 72 ^o^C | Shaw et al. 2007 |
|  | trnL^UAG^ | R | CTGCTTCCTAAGAGCAGCGT |  |  |  |
| *trnL*-gene intron | trnL. c | F | CGAAATCGGTAGACGCTACG | 1×PCR buffer, 0.05 U Taq polymerase, 0.6 mM MgCl_2_, 1.0 mM of each dNTP, 0.25 mM of each primer and 1 μL of template DNA | 5 min at 94 ^o^C, 30× (30 s at 94 ^o^C, 30 s at 54 ^o^C, 60 s at 72 ^o^C), 10 min at 72 ^o^C | Taberlet et al. 1991 |
|  | trnL. d | R | GGGGATAGAGGGACTTGA AC |  |  |  |
| *trnL-trnF* | trnL. e | F | GGTTCAAGTCCCTCTATCCC | 1×PCR buffer, 0.05 U Taq polymerase, 0.6 mM MgCl_2_, 1.0 mM of each dNTP, 0.25 mM of each primer and 1 μL of template DNA | 5 min at 94 ^o^C, 30× (30 s at 94 ^o^C, 30 s at 50 ^o^C, 60 s at 72 ^o^C), 10 min at 72 ^o^C | Taberlet et al. 1991 |
|  | trnL. f | R | ATTTGAACTGGTGACACG AG |  |  |  |

CGAAATCGGTAGACGCTACG

**Additional references**

**Kress WJ, Erickson DL.** **2007**. A two-locus global DNA barcode for land plants: the coding *rbcl* gene complements the non-coding *trnH-psbA* spacer region. *PLOS ONE,* **2**: e508.

**Shaw J, Lickey EB, Schilling EE, Small RL.** **2007**. Comparison of whole chloroplast genome sequences to choose noncoding regions for phylogenetic studies in angiosperms: the tortoise and the hare III. *American journal of botany,* **94**: 275-288.

**Whitten WM, Williams NH, Chase MW.** **2000**. Subtribal and generic relationships of Maxillarieae (Orchidaceae) with emphasis on Stanhopeinae: combined molecular evidence. *American Journal of Botany,* **87**: 1842-1856.

Supplementary Table S3. Spatial analysis of molecular variation (SAMOVA) of ITS sequence data across British *Euphrasia* populations.

| *K* | *Fct* | *Fst* | *Fsc* | Group composition |
| --- | --- | --- | --- | --- |
| 2 | 0.827 | 0.868 | 0.235 | {*E. anglica*_SW, *E. anglica*_W, *E. rivularis*_W, *E. rostkoviana*_W, *E. vigursii*_SW} {*E. arctica*_SW, *E. arctica*_W, *E. cambrica*_W, *E. campbelliae*_S, *E. confusa*_S, *E. confusa*_SW, *E. confusa*_W, *E. foulaensis*_S, *E. frigida*_S, *E. heslop-harrisonii*_S, *E. marshallii*_S, *E. micrantha*_SW, *E. micrantha*_W, *E. nemorosa*_S, *E. nemorosa*_SW, *E. nemorosa*_W, *E. ostenfeldii*_S, *E. ostenfeldii*_W, *E. pseudokerneri*_W, *E. scottica*_S, *E. scottica*_W, *E. tetraquetra*_SW, *E. tetraquetra*_W} |
| 3 | 0.815 | 0.860 | 0.246 | {*E. anglica*_SW, *E. anglica*_W, *E. vigursii*_SW} {*E. arctica*_SW, *E. arctica*_W, *E. cambrica*_W, *E. campbelliae*_S, *E. confusa*_S, *E. confusa*_SW, *E. confusa*_W, *E. foulaensis*_S, *E. frigida*_S, *E. heslop-harrisonii*_S, *E. marshallii*_S, *E. micrantha*_SW, *E. micrantha*_W, *E. nemorosa*_S, *E. nemorosa*_SW, *E. nemorosa*_W, *E. ostenfeldii*_S, *E. ostenfeldii*_W, *E. pseudokerneri*_W, *E. scottica*_S, *E. scottica*_W, *E. tetraquetra*_SW, *E. tetraquetra*_W} {*E. rivularis*_W, *E. rostkoviana*_W} |
| 4 | 0.819 | 0.854 | 0.191 | {*E. anglica*_SW, *E. anglica*_W, *E. rivularis*_W, *E.vigursii*_SW} {*E. arctica*_SW, *E. arctica*_W, *E. cambrica*_W,  *E. campbelliae*_S, *E. confusa*_S, *E. confusa*_SW, *E. confusa*_W, *E. foulaensis*_S, *E. frigida*_S, *E. heslop-harrisonii*_S,  *E. marshallii*_S, *E. micrantha*_SW, *E. micrantha*_W, *E. nemorosa*_S, *E. nemorosa*_SW, *E. nemorosa*_W, *E. ostenfeldii*_W,  *E. pseudokerneri*_W, *E. scottica*_S, *E. scottica*_W, *E. tetraquetra*_SW, *E. tetraquetra*_W} {*E. ostenfeldii*_S}  {*E. rostkoviana*_W} |
| 5 | 0.839 | 0.844 | 0.026 | {*E. anglica*_SW, *E. anglica*_W, *E. rostkoviana*_W, *E.vigursii*_SW}{*E. arctica*_SW, *E. arctica*_W, *E. cambrica*_W, *E. campbelliae*_S, *E. confusa*_S, *E. confusa*_SW, *E. confusa*_W, *E. foulaensis*_S, *E. frigida*_S, *E. heslop-harrisonii*_S, *E. marshallii*_S, *E. micrantha*_SW, *E. micrantha*_W, *E. nemorosa*_S, *E. nemorosa*_SW, *E. nemorosa*_W, *E. ostenfeldii*_W, *E. pseudokerneri*_W, *E. scottica*_S, *E. tetraquetra*_SW, *E. tetraquetra*_W} {*E. ostenfeldii*_S} {*E. rivularis*_W} {*E. scottica*_W} |
| 6 | 0.819 | 0.826 | 0.039 | {*E. anglica*_SW, *E. anglica*_W, *E.vigursii*_SW} {*E. arctica*_SW, *E. arctica*_W, *E. cambrica*_W, *E. campbelliae*_S, *E. confusa*_S, *E. confusa*_SW, *E. confusa*_W, *E. foulaensis*_S, *E. frigida*_S, *E. heslop-harrisonii*_S, *E. marshallii*_S, *E. micrantha*_SW, *E. micrantha*_W, *E. nemorosa*_S, *E. nemorosa*_SW, *E. nemorosa*_W, *E. ostenfeldii*_W, *E. pseudokerneri*_W, *E. scottica*_S, *E. tetraquetra*_W} { *E. tetraquetra*_SW } {*E. ostenfeldii*_S} {*E. rostkoviana*_W, *E. rivularis*_W } {*E. scottica*_W} |
| 7 | 0.839 | 0.837 | -0.016 | {*E. anglica*_SW, *E. rivularis*_W, *E.vigursii*_SW} {*E. anglica*_W} {*E. arctica*_SW, *E. arctica*_W, *E. cambrica*_W, *E. campbelliae*_S, *E. confusa*_S, *E. confusa*_SW, *E. confusa*_W, *E. foulaensis*_S, *E. frigida*_S, *E. heslop-harrisonii*_S, *E. marshallii*_S, *E. micrantha*_SW, *E. micrantha*_W, *E. nemorosa*_S, *E. nemorosa*_W, *E. ostenfeldii*_W, *E. pseudokerneri*_W, *E. scottica*_S, *E. tetraquetra*_SW, *E. tetraquetra*_W} {*E. nemorosa*_SW} {*E. ostenfeldii*_S} {*E. rostkoviana*_W} {*E. scottica*_W} |
| 8 | 0.819 | 0.817 | -0.009 | {*E. anglica*_SW, *E. anglica*_W, *E.vigursii*_SW} {*E. arctica*_SW} {*E. arctica*_W, *E. campbelliae*_S, *E. confusa*_S, *E. confusa*_SW, *E. confusa*_W, *E. foulaensis*_S, *E. frigida*_S, *E. heslop-harrisonii*_S, *E. marshallii*_S, *E. micrantha*_SW, *E. micrantha*_W, *E. nemorosa*_S, *E. nemorosa*_SW, *E. nemorosa*_W, *E. ostenfeldii*_W, *E. pseudokerneri*_W, *E. scottica*_S, *E. tetraquetra*_SW, *E. tetraquetra*_W} {*E. cambrica*_W} {*E. ostenfeldii*_S} {*E. rivularis*_W} {*E. rostkoviana*_W} {*E. scottica*_W} |
| 9 | 0.822 | 0.812 | -0.059 | {*E. anglica*_SW, *E.vigursii*_SW} {*E. anglica*_W} {*E. arctica*_SW, *E. arctica*_W, *E. cambrica*_W, *E. campbelliae*_S, *E. confusa*_S, *E. confusa*_W, *E. foulaensis*_S, *E. frigida*_S, *E. heslop-harrisonii*_S, *E. marshallii*_S, *E. micrantha*_SW, *E. nemorosa*_S, *E. nemorosa*_SW, *E. nemorosa*_W, *E. ostenfeldii*_W, *E. pseudokerneri*_W, *E. scottica*_S, *E. tetraquetra*_SW, *E. tetraquetra*_W} {*E. confusa*_SW} {*E. micrantha*_W} {*E. ostenfeldii*_S} {*E. rivularis*_W} {*E. rostkoviana*_W} {*E. scottica*_W} |
| 10 | 0.812 | 0.791 | -0.113 | {*E. anglica*_SW, *E. anglica*_W, *E.vigursii*_SW} {*E. arctica*_SW, *E. arctica*_W, *E. campbelliae*_S, *E. confusa*_S, *E. foulaensis*_S,  *E. frigida*_S, *E. heslop-harrisonii*_S, *E. marshallii*_S, *E. micrantha*_SW, *E. micrantha*_W, *E. nemorosa*_S, *E. nemorosa*_SW,  *E. nemorosa*_W, *E. ostenfeldii*_W, *E. scottica*_S, *E. tetraquetra*_SW, *E. tetraquetra*_W} {*E. cambrica*_W} {*E. confusa*_SW} {*E. confusa*_W} {*E. ostenfeldii*_S} {*E. pseudokerneri*_W} {*E. rivularis*_W} {*E. rostkoviana*_W} {*E. scottica*_W} |

*K* refers to the number of predefined groups used in the analyses. All tests for differentiation are significant at *P* < 0.001. Species name followed by underline with W, SW or S means that one population (from one to five samples per population) from Wales, Southwest of England or Scotland.

Supplementary Table S4. Plastid haplotype frequencies across British *Euphrasia* species.

| Taxa | No. | Haplotype | Nucleotide diversity |
| --- | --- | --- | --- |
| *E. anglica*_SW | 4 | H1(1), H2(3) | 0.0017 |
| *E. anglica_*W | 3 | H2(1), H3(1), H4(1) | 0.0018 |
| *E. arctica_*S | 3 | H5(1), H6(1), H7(1) | 0.0041 |
| *E. arctica_*SW | 2 | H4(1), H5(1) | 0.0028 |
| *E. arctica_*W | 2 | H3(1), H6(1) | 0.0034 |
| *E. arctica x confusa*_S | 1 | H8(1) | 0 |
| *E. arctica x foulaensis_*S | 1 | H9(1) | 0 |
| *E. arctica x micrantha_*S | 3 | H5(2), H10(1) | 0.005 |
| *E. arctica x nemorosa_*S | 2 | H5(1), H7(1) | 0.0055 |
| *E. arctica x rostkoviana_*S | 3 | H7(1), H11(1), H12(1) | 0.0073 |
| *E. cambrica_*W | 3 | H1(3) | 0 |
| *E. campbelliae_*S | 3 | H1(3) | 0 |
| *E. confusa_*S | 4 | H4(3), H5(1) | 0.0014 |
| *E. confusa_*SW | 3 | H2(1), H13(1), H14(1) | 0.0023 |
| *E. confusa* W | 3 | H2(2), H15(1) | 0.0018 |
| *E. confusa x micrantha_*S | 2 | H2(1), H16(1) | 0.0055 |
| *E. fharaidensis_*S | 3 | H6(1), H10(1), H11(1) | 0.0046 |
| *E. foulaensis_*S | 6 | H5(1), H7(1), H10(2), H17(1), H18(1) | 0.0044 |
| *E. foulaensis x marshllii_*S | 2 | H1(1), H6(1) | 0.0041 |
| *E. foulaensis x nemorosa*_S | 1 | H1(1) | 0 |
| *E. foulaensis x ostenfeldii_*S | 1 | H4(1) | 0 |
| *E. frigida_*S | 5 | H1(1), H4(2), H19(1), H20(1) | 0.001 |
| *E. heslop-harrisoni*i_S | 6 | H1(1), H2(1), H4(1), H5(1), H6(2) | 0.003 |
| *E. marshllii_*S | 3 | H5(2), H21(1) | 0.0041 |
| *E. marshallii x micrantha_*S | 2 | H11(1), H21(1) | 0.0062 |
| *E. micrantha_*S | 5 | H2(1), H4(1), H6(1), H7(1), H22(1) | 0.003 |
| *E. micrantha_*SW | 3 | H5(2), H23(1) | 0.0032 |
| *E. micrantha_*W | 3 | H4(1), H12(1), H24(1) | 0.0041 |
| *E. micrantha x nemorosa_*SW | 1 | H5(1) | 0 |
| *E. micrantha x scottica*_S | 5 | H1(1), H5(1), H6(2), H7(1) | 0.0036 |
| *E. nemorosa_*S | 3 | H4(1), H5(2) | 0.0018 |
| *E. nemorosa_*SW | 2 | H4(2) | 0 |
| *E. nemorosa_*W | 3 | H25(1), H26(1), H27(1) | 0.0009 |
| *E. nemorosa x tetraquetra_*SW | 1 | H4(1) | 0 |
| *E. ostenfeldii_*S | 5 | H4(1), H6(2), H28(1), H29(1) | 0.0043 |
| *E. ostenfeldii_*W | 1 | H1(1) | 0 |
| *E. pseudokerneri_*W | 3 | H30(1), H31(1), H32(1) | 0.0028 |
| *E. rivularis_*W | 3 | H1(1), H20(1), H33(1) | 0.0028 |
| *E. rostkoviana_*W | 2 | H2(1), H4(1) | 0.0021 |
| *E. rotundifolia_*S | 1 | H17(1) | 0 |
| *E. scottica_*S | 3 | H34(3) | 0 |
| *E. scottica_*W | 3 | H4(3) | 0 |
| *E. tetraquetra_*SW | 3 | H2(1), H5(2) | 0.0032 |
| *E. tetraquetra_*W | 3 | H35(1), H36(1), H37(1) | 0.0037 |
| *E. tetraquetra x vigursii_*SW | 2 | H2(1), H4(1) | 0.0021 |
| *E. vigursii*_SW | 4 | H2(3), H38(1) | 0.0007 |
| Total | 130 |  | 0.0035 |

Supplementary Table S5. Spatial analysis of molecular variation (SAMOVA) of plastid sequence data across British *Euphrasia* populations.

| *K* | *Fct* | *Fst* | *Fsc* | Group composition |
| --- | --- | --- | --- | --- |
| 2 | 0.322 | 0.526 | 0.300 | {*E. anglica*_SW, *E. anglica*_W, *E. arctica*_SW, *E. arctica*_W, *E. cambrica*_W, *E. campbelliae*_S, *E. confusa*_S, *E. confusa*_SW, *E. confusa*_W, *E. frigida*_S, *E. heslopharrisonii*_S, *E. marshallii*_S, *E. micrantha*_SW, *E. micrantha*_W, *E. nemorosa*_S, *E. nemorosa*_SW, *E. nemorosa*_W, *E. ostenfeldii*_S, *E. ostenfeldii*_W, *E. pseudokerneri*_W, *E. rivularis*_W, *E. rostkoviana*_W, *E. scottica*_S, *E. scottica*_W, *E. tetraquetra*_SW, *E. tetraquetra*_W, *E. vigursii*_SW} {*E. foulaensis*_S} |
| 3 | 0.361 | 0.430 | 0.108 | {*E. anglica*_SW, *E. vigursii*_SW} {*E. anglica*_W, *E. arctica*_SW, *E. arctica*_W, *E. cambrica*_W, *E. campbelliae*_S, *E. confusa*_S, *E. confusa*_SW, *E. confusa*_W, *E. frigida*_S, *E. micrantha*_W, *E. nemorosa*_SW, *E. nemorosa*_W, *E. ostenfeldii*_S, *E. ostenfeldii*_W, *E. pseudokerneri*_W, *E. rivularis*_W, *E. rostkoviana*_W, *E. scottica*_S, *E. scottica*_W, *E. tetraquetra*_W} {*E. foulaensis*_S, *E. heslopharrisonii*_S, *E. marshallii*_S, *E. micrantha*_SW, *E. nemorosa*_S, *E. tetraquetra*_SW} |
| 4 | 0.344 | 0.437 | 0.142 | {*E. anglica*_SW, *E. confusa*_SW, *E. confusa*_W, *E. vigursii*_SW} {*E. anglica*_W, *E. arctica*_SW, *E. arctica*_W, *E. cambrica*_W, *E. campbelliae*_S, *E. confusa*_S, *E. frigida*_S, *E. heslopharrisonii*_S, *E. micrantha*_W, *E. nemorosa*_S, *E. nemorosa*_SW, *E. nemorosa*_W, *E. ostenfeldii*_S, *E. ostenfeldii*_W, *E. pseudokerneri*_W, *E. rivularis*_W, *E. rostkoviana*_W, *E. scottica*_S, *E. scottica*_W, *E. tetraquetra*_W} {*E. foulaensis*_S, *E. marshallii*_S} {*E. micrantha*_SW, *E. tetraquetra*_SW} |
| 5 | 0.385 | 0.446 | 0.100 | {*E. anglica*_SW, *E. confusa*_SW, *E. confusa*_W, *E. vigursii*_SW} {*E. anglica*_W, *E. arctica*_SW, *E. arctica*_W, *E. confusa*_S, *E. frigida*_S, *E. heslopharrisonii*_S, *E. marshallii*_S, *E. micrantha*_SW, *E. micrantha*_W, *E. nemorosa*_S, *E. nemorosa*_SW, *E. nemorosa*_W, *E. pseudokerneri*_W, *E. rivularis*_W, *E. rostkoviana*_W, *E. scottica*_S, *E. scottica*_W, *E. tetraquetra*_SW, *E. tetraquetra*_W} {*E. cambrica*_W, *E. campbelliae*_S, *E. ostenfeldii*_W} {*E. foulaensis*_S} {*E. ostenfeldii*_S} |
| 6 | 0.387 | 0.387 | -0.005 | {*E. anglica*_SW, *E. confusa*_SW, *E. confusa*_W, *E. rostkoviana*_W, *E. vigursii*_SW} {*E. anglica*_W, *E. cambrica*_W, *E. campbelliae*_S, *E. confusa*_S, *E. frigida*_S, *E. nemorosa*_SW, *E. ostenfeldii*_S, *E. ostenfeldii*_W, *E. rivularis*_W, *E. scottica*_S, *E. scottica*_W, *E. tetraquetra*_W} {*E. arctica*_SW, *E. arctica*_W, *E. micrantha*_SW, *E. pseudokerneri*_W, *E. tetraquetra*_SW} {*E. foulaensis*_S, *E. heslopharrisonii*_S, *E. marshallii*_S, *E. nemorosa*_S} {*E. micrantha*_W} {*E. nemorosa*_W} |
| 7 | 0.388 | 0.379 | -0.015 | {*E. anglica*_SW, *E. anglica*_W, *E. confusa*_SW, *E. confusa*_W, *E. rostkoviana*_W} {*E. arctica*_SW, *E. arctica*_W, *E. micrantha*_SW, *E. micrantha*_W, *E. pseudokerneri*_W, *E. tetraquetra*_SW} {*E. cambrica*_W, *E. campbelliae*_S, *E. confusa*_S, *E. frigida*_S, *E. nemorosa*_SW, *E. ostenfeldii*_S, *E. ostenfeldii*_W, *E. rivularis*_W, *E. scottica*_S, *E. scottica*_W, *E. tetraquetra*_W} {*E. foulaensis*_S, *E. heslopharrisonii*_S} {*E. marshallii*_S, *E. nemorosa*_S} {*E. nemorosa*_W} {*E. vigursii*_SW} |
| 8 | 0.422 | 0.380 | -0.073 | {*E. anglica*_SW, *E. anglica*_W, *E. confusa*_SW, *E. confusa*_W, *E. rostkoviana*_W} {*E. arctica*_SW, *E. arctica*_W, *E. heslopharrisonii*_S, *E. marshallii*_S, *E. micrantha*_SW, *E. nemorosa*_S, *E. tetraquetra*_SW} {*E. cambrica*_W, *E. campbelliae*_S, *E. confusa*_S, *E. frigida*_S, *E. nemorosa*_SW, *E. ostenfeldii*_W, *E. rivularis*_W, *E. scottica*_S, *E. scottica*_W, *E. tetraquetra*_W} {*E. foulaensis*_S} {*E. micrantha*_W, *E. pseudokerneri*_W } {*E. nemorosa*_W} {*E. ostenfeldii*_S} {*E. vigursii*_SW} |
| 9 | 0.450 | 0.370 | -0.144 | {*E. anglica*_SW, *E. confusa*_SW, *E. vigursii*_SW} {*E. anglica*_W, *E. confusa*_S, *E. frigida*_S, *E. nemorosa*_SW, *E. rivularis*_W, *E. scottica*_S, *E. scottica*_W, *E. tetraquetra*_W} {*E. arctica*_SW, *E. arctica*_W, *E. heslopharrisonii*_S, *E. marshallii*_S, *E. micrantha*_SW, *E. nemorosa*_S, *E. tetraquetra*_SW} {*E. cambrica*_W, *E. campbelliae*_S, *E. ostenfeldii*_W} {*E. confusa*_W, *E. rostkoviana*_W} {*E. foulaensis*_S} {*E. micrantha*_W, *E. pseudokerneri*_W} {*E. nemorosa*_W} {*E. ostenfeldii*_S} |
| 10 | 0.450 | 0.360 | -0.164 | {*E. anglica*_SW, *E. anglica*_W, *E. confusa*_SW, *E. confusa*_W, *E. rivularis*_W, *E. rostkoviana*_W} {*E. arctica*_SW, *E. arctica*_W, *E. micrantha*_SW, *E. tetraquetra*_SW} {*E. cambrica*_W, *E. campbelliae*_S, *E. ostenfeldii*_W} {*E. confusa*_S, *E. frigida*_S, *E. nemorosa*_SW, *E. scottica*_S, *E. scottica*_W, *E. tetraquetra*_W} {*E. foulaensis*_S, *E. heslopharrisonii*_S} {*E. marshallii*_S, *E. nemorosa*_S} {*E. micrantha*_W, *E. pseudokerneri*_W} {*E. nemorosa*_W} {*E. ostenfeldii*_S} {*E. vigursii*_SW} |

*K* refers to the number of predefined groups used in the analyses. All tests for differentiation are significant at *P* < 0.001. Species name followed by underline with W, SW or S means that one population (from one to five samples per population) from Wales, Southwest of England or Scotland.

　　 Table S6. The distribution of matK haplotypes for populations of British *Euphrasia*. The cpDNA(n) column indicates the sample sizes.

|  | CpDNA  (n) | H1 | H2 | H3 | H4 | H5 | H6 | H7 | H8 | H9 | H1 | H11 | H12 | H13 | H14 | H15 | H16 | H17 |
| --- | --- | --- | --- | --- | --- | --- | --- | --- | --- | --- | --- | --- | --- | --- | --- | --- | --- | --- |
| *E. anglica_*SW | 4 | 1 | 3 |  |  |  |  |  |  |  |  |  |  |  |  |  |  |  |
| *E. anglica_*W | 3 |  | 2 | 1 |  |  |  |  |  |  |  |  |  |  |  |  |  |  |
| *E. arctica_*S | 3 |  | 1 |  | 2 |  |  |  |  |  |  |  |  |  |  |  |  |  |
| *E. arctica_*SW | 2 |  | 1 |  | 1 |  |  |  |  |  |  |  |  |  |  |  |  |  |
| *E. arctica_*W | 2 |  |  | 1 | 1 |  |  |  |  |  |  |  |  |  |  |  |  |  |
| *E. arctica* x *confusa_*S | 1 |  | 1 |  |  |  |  |  |  |  |  |  |  |  |  |  |  |  |
| *E. arctica* x *foulaensis_*S | 1 |  |  |  |  | 1 |  |  |  |  |  |  |  |  |  |  |  |  |
| *E. arctica* x *micrantha_*S | 3 |  | 1 |  | 2 |  |  |  |  |  |  |  |  |  |  |  |  |  |
| *E. arctica* x *nemorosa_*S | 2 |  | 1 |  | 1 |  |  |  |  |  |  |  |  |  |  |  |  |  |
| *E. arctica* x *rostkoviana_*S | 3 |  | 2 |  |  |  | 1 |  |  |  |  |  |  |  |  |  |  |  |
| *E. cambrica_*W | 3 | 3 |  |  |  |  |  |  |  |  |  |  |  |  |  |  |  |  |
| *E. campbelliae_*S | 3 | 3 |  |  |  |  |  |  |  |  |  |  |  |  |  |  |  |  |
| *E. confusa_*S | 4 |  | 3 |  | 1 |  |  |  |  |  |  |  |  |  |  |  |  |  |
| *E. confusa_*SW | 3 |  | 2 |  |  |  |  | 1 |  |  |  |  |  |  |  |  |  |  |
| *E. confusa* W | 3 |  | 2 | 1 |  |  |  |  |  |  |  |  |  |  |  |  |  |  |
| *E. confusa* x *micrantha_*S | 2 |  | 2 |  |  |  |  |  |  |  |  |  |  |  |  |  |  |  |
| *E. fharaidensis_*S | 3 |  | 2 |  | 1 |  |  |  |  |  |  |  |  |  |  |  |  |  |
| *E. foulaensis_*S | 6 |  | 3 |  | 3 |  |  |  |  |  |  |  |  |  |  |  |  |  |
| *E. foulaensis* x *marshllii_*S | 2 | 1 |  |  | 1 |  |  |  |  |  |  |  |  |  |  |  |  |  |
| *E. foulaensis* x *nemorosa_*S | 1 | 1 |  |  |  |  |  |  |  |  |  |  |  |  |  |  |  |  |
| *E. foulaensis* x *ostenfeldii_*S | 1 |  | 1 |  |  |  |  |  |  |  |  |  |  |  |  |  |  |  |
| *E. frigida_*S | 5 | 1 | 4 |  |  |  |  |  |  |  |  |  |  |  |  |  |  |  |
| *E. heslop-harrisonii_*S | 6 | 1 | 2 |  | 3 |  |  |  |  |  |  |  |  |  |  |  |  |  |
| *E. marshllii_*S | 3 | 1 |  |  | 2 |  |  |  |  |  |  |  |  |  |  |  |  |  |
| *E. marshallii* x *micrantha_*S | 2 | 1 | 1 |  |  |  |  |  |  |  |  |  |  |  |  |  |  |  |
| *E. micrantha_*S | 5 |  | 4 |  | 1 |  |  |  |  |  |  |  |  |  |  |  |  |  |
| *E. micrantha_*SW | 3 |  | 1 |  | 2 |  |  |  |  |  |  |  |  |  |  |  |  |  |
| *E. micrantha_*W | 3 |  | 1 |  |  |  | 1 |  | 1 |  |  |  |  |  |  |  |  |  |
| *E. micrantha* x *nemorosa_*SW | 1 |  |  |  | 1 |  |  |  |  |  |  |  |  |  |  |  |  |  |
| *E. micrantha* x *scottica_*S | 5 | 1 | 1 |  | 3 |  |  |  |  |  |  |  |  |  |  |  |  |  |
| *E. nemorosa_*S | 3 |  | 1 |  | 2 |  |  |  |  |  |  |  |  |  |  |  |  |  |
| *E. nemorosa_*SW | 2 |  | 2 |  |  |  |  |  |  |  |  |  |  |  |  |  |  |  |
| *E. nemorosa_*W | 3 |  |  |  |  |  |  |  |  | 1 | 1 | 1 |  |  |  |  |  |  |
| *E. nemorosa* x *tetraquetra_*SW | 1 |  | 1 |  |  |  |  |  |  |  |  |  |  |  |  |  |  |  |
| *E. ostenfeldii_*S | 5 | 1 | 1 |  | 2 |  |  |  |  |  |  |  | 1 |  |  |  |  |  |
| *E. ostenfeldii_*W | 1 | 1 |  |  |  |  |  |  |  |  |  |  |  |  |  |  |  |  |
| *E. pseudokerneri_*W | 3 |  |  |  | 1 |  |  |  |  |  |  |  |  | 1 | 1 |  |  |  |
| *E. rivularis_*W | 3 | 1 | 2 |  |  |  |  |  |  |  |  |  |  |  |  |  |  |  |
| *E. rostkoviana_*W | 2 |  | 2 |  |  |  |  |  |  |  |  |  |  |  |  |  |  |  |
| *E. rotundifolia_*S | 1 |  |  |  | 1 |  |  |  |  |  |  |  |  |  |  |  |  |  |
| *E. scottica_*S | 3 |  | 3 |  |  |  |  |  |  |  |  |  |  |  |  |  |  |  |
| *E. scottica_*W | 3 |  | 3 |  |  |  |  |  |  |  |  |  |  |  |  |  |  |  |
| *E. tetraquetra_*SW | 3 |  | 1 |  | 2 |  |  |  |  |  |  |  |  |  |  |  |  |  |
| *E. tetraquetra_*W | 3 |  | 1 |  |  |  |  |  |  |  |  |  |  |  |  | 1 | 1 |  |
| *E. tetraquetra* x *vigursii_*SW | 2 |  | 2 |  |  |  |  |  |  |  |  |  |  |  |  |  |  |  |
| *E. vigursii_*SW | 4 |  | 3 |  |  |  |  |  |  |  |  |  |  |  |  |  |  | 1 |
| Total | 130 | 17 | 63 | 3 | 33 | 1 | 2 | 1 | 1 | 1 | 1 | 1 | 1 | 1 | 1 | 1 | 1 | 1 |
|  |  |  |  |  |  |  |  |  |  |  |  |  |  |  |  |  |  |  |

Table S7. Hierarchical analysis of molecular variance (AMOVA) for *matK* sequenced in British *Euphrasia* populations. Analyses performed between (A) species, (B) 3 geographic locations (Wales, South-West England, Scotland, (C) diploids and tetraploids. Number in parentheses are the results only including species (excluding hybrids). d.f. = Degrees of freedom. ***P* < 0.001;**P* < 0.05, ^ns^ nonsignificant.

| **Source of variation** | **d.f.** | **Percentage of variation** |
| --- | --- | --- |
| **(A) Taxa** |  |  |
| **Between taxa** | 33(19) | 13.37 ^ns^(16.84^**^) |
| **Within taxa** | 96(83) | 86.63(83.16) |
| **(B) Location** |  |  |
| **Between regions** | 2(2) | 5.46^*^ (4.47^*^) |
| **Within regions** | 127(102) | 94.54(95.53) |
| **(C) Ploidy** |  |  |
| **Between ploidy groups** | 1(1) | 7.05^*^ (5.94^*^) |
| **Within diploids and tetraploids** | 128(101) | 92.95(94.06) |
